# Protonation-Induced Chemical Transformations in Mass Spectrometry: Implications for Detecting Complex Organics on Icy Moons

**DOI:** 10.1021/acsearthspacechem.5c00363

**Published:** 2026-02-19

**Authors:** Lucía Hortal Sánchez, Maryse Napoleoni, Ernesto Brunet, Fabian Klenner, Thomas R. O’Sullivan, Mirandah Ackley, Gregoire Danger, Bernd Abel, Nozair Khawaja, Frank Postberg

**Affiliations:** † Institut für Geologische Wissenschaften, 9166 Freie Universität Berlin, Berlin 12249, Germany; ‡ Department of Organic Chemistry, 16722 Universidad Autónoma de Madrid, Madrid 28049, Spain; § Department of Earth and Space Sciences, University of Washington, Seattle, Washington 98195, United States; ∥ Department of Earth and Planetary Sciences, University of California, Riverside, California 92521, United States; ⊥ 128791 Aix Marseille Univ, CNRS, Institut Origines, PIIM, Marseille 13013, France; # Institute of Chemical Technology, 28395 University Leipzig, Leipzig 04103, Germany; ∇ Department Space Chemistry and Technology, J. Heyrovsky Institute of Physical Chemistry, Czech Academy of Sciences, Praha 182 23, Czech Republic

**Keywords:** reactivity, organics, laser, icy moon, impact ionization, mass-spectrometry, enceladus, europa, protonation, transformations

## Abstract

Impact ionization mass spectrometers, such as Cassini’s Cosmic Dust Analyzer, are capable of detecting macromolecular organic compounds in ice grains ejected from icy moons such as Enceladus and Europa. The identification of their chemical features relies on laboratory analogue experiments that replicate ice grain impact ionization mass spectra, such as the laser-induced liquid beam ion desorption (LILBID) technique. Both space-borne instruments and analogue experiments require a deeper understanding of measurement-associated processes affecting mass spectral features, and in particular protonation-induced chemical transformations (PICTs). Here, we investigate the molecule amygdalin (C_20_H_27_NO_11_) as a model high-mass, complex organic compound using LILBID to determine its mass spectral fingerprint. Our results show that amygdalin undergoes unexpected PICTs enabled by the high laser energy input upon measurement. The chemical transformations are promoted by the proton-rich environment created upon the disintegration of the water matrix. This reactivity is distinct from other well-characterized phenomena affecting analytes under LILBID conditions (e.g., fragmentation). Protonation triggers reactivity in amygdalin’s nitrile group resulting in multiple products that appear as characteristic molecular ions. Nuclear magnetic resonance spectroscopy experiments confirm that this reactivity occurs under LILBID measurement, not in solution prior to desorption. Compounds with similar functional groups (e.g., amide or ketone) could, in principle, also be subject to PICTs. PICTs could also occur in space during space-borne impact ionization, potentially complicating the identification of analytes embedded in ice grains. Our work builds toward a better understanding of the effects of PICTs in the detection of organic compounds with impact ionization mass spectrometry.

## Graphical abstract

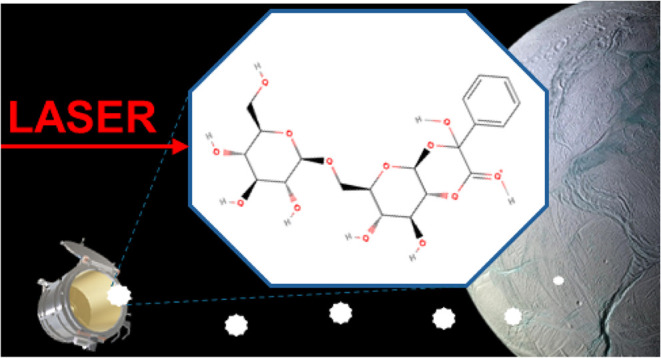

## Introduction

Icy ocean moons, especially Europa and Enceladus, are considered among the most habitable planetary bodies in the solar system beyond Earth. Within subsurface oceans in the interior of these moons, hydrothermal activity can occur at the seafloor,^1,2^ hinting at high astrobiological potential. This has in turn, lead to interest from the major space agencies in exploratory missions.^3–7^ Ice grains containing subsurface-originating material are ejected from these moons by cryovolcanic plumes or micrometeorite bombardment.^8–11^ These ice grains can be sampled and analyzed by impact ionization mass spectrometers onboard spacecraft.^12^ The Cassini-Huygens mission characterized ice grains emitted from the Saturnian moon Enceladus in such fashion with its Cosmic Dust Analyzer (CDA^13^). NASA’s recently launched Europa Clipper mission is also equipped with an impact ionization mass spectrometer—the SUrface Dust Analyzer (SUDA^12^)—which is built upon CDA heritage and offers significant improvements in mass resolution and sensitivity. SUDA also has the capacity to carry out dual polarity measurements (i.e., cations and anions)—whereas CDA could only detect cations.^13^ New generations of advanced impact ionization mass spectrometers, such as the High Ice Flux Instrument (HIFI), are currently being developed for future Enceladus missions.^6^

Impact ionization mass spectrometers such as CDA and SUDA, are designed to analyze dust and ice particles they encounter in flight. Incident particles strike the metal plate at the bottom of the instrument’s aperture at speeds >1 km/s and form impact clouds comprised of electrons, both neutral and charged species, neutral molecules, and macroscopic fragments. The ions created are almost exclusively singly charged.^14–16^ Ions are then separated by polarity and accelerated toward a detector. Their arrival times, dictated by their mass, are translated into time-of-flight mass spectrometric data. Since the energy input for ionization has a kinetic origin, the extent of fragmentation of a given organic molecule(s) embedded within an ice grain is strongly coupled with the spacecraft encounter speed,^17^ which, for Cassini, varied between 2 and 30 km/s.^18^

The interpretation of CDA mass spectra has characterized key features of Enceladus’ ocean, including composition, salinity, the presence of water-rock interactions, pH ranges, constraints on redox potential and the existence of physicochemical disequilibria at the seafloor, which is also the site of ongoing hydrothermal activity.^1,10,19–22^ Moreover, at least five of the six elements (CHONP, with a tentative detection of S) considered essential to life on Earth have been detected alongside a suite of organic compounds.^2,23–26^ These organics exhibit diverse chemical properties, ranging in size from simple molecules to complex macromolecular structures, and comprise many different moieties and functional groups.^23,25,26^ The reliable interpretation of data from CDA and other spaceborne mass spectrometers is made possible by means of analogue experiments carried out on Earth using the laser-induced liquid beam ion desorption (LILBID) technique.

In space, solid material (ice grains) is analyzed by impact ionization instruments while LILBID analysis is performed by the desorption of a liquid water matrix^17^ due to the technical challenges associated with accelerating ice grains. Both techniques, however, exhibit charge exchange phenomena in the gas phase and result in mostly singly charged particles.^15,16,27^ Different impact speeds of ice grains upon the instrument’s target in space can be simulated in LILBID by changing the laser’s power density and the delayed extraction time of ions of the mass spectrometer (see Methods).^17^

Previous experiments with LILBID have been performed with a variety of organic compounds, relevant for the habitability of icy ocean moons.^25,26,28–36^ The data obtained enables spectral appearance predictions of putative chemical components transported in the ice grains emitted from these moons.^28,32–34^ Isomeric compounds have also been investigated with LILBID, with a special interest in spectral differences derived from the positioning of their functional groups.^30^ These experiments relate a wide variety of functional groups and, more generally, molecules to their spectral features, thereby providing a reference for the interpretation of data obtained by impact ionization instruments. All experimental results from LILBID are retained in a spectral database^37^ to facilitate comprehensive and quick interpretation of mass spectra obtained by space missions.

Elucidating the identity of the macromolecular organics present in a significant portion of ice grains liberated from Enceladus,^23^ would shed light into the habitability and astrobiological potential of this and other icy ocean worlds. Some of these high mass organic compounds detected by CDA present characteristic spectral features indicative of a variety of functional groups with aromatic and aliphatic moieties, and N- and O-bearing functional groups. This underscores the need for laboratory analogue experiments investigating large (>200 u) complex organics encompassing a variety of moieties and heteroatom-containing functional groups.

The present work investigates the spectral appearance of the molecule amygdalin (C_20_H_27_NO_11_, mass of 457.43 g/mol) with LILBID. Amygdalin was selected due to its variety of moieties, each presenting a range of chemical characteristics (e.g., aromatic, aliphatic, polar, nonpolar, electrophilic, nucleophilic, etc.) relevant for the investigation of high-mass organics detected in Enceladean ice grains. The recorded LILBID spectra show that amygdalin reacts during the matrix desorption process, via both hydrolysis and condensation reactions. Nuclear Magnetic Resonance (NMR) spectroscopy was used to verify the observed reactivity of amygdalin, and the details of the reaction pathways were investigated.

## Experimental Section

### Amygdalin Samples

Amygdalin (C_20_H_27_NO_11_; mass = 457.43 g/mol and 97% purity, obtained from TCI) is an aromatic cyanogenic glycoside that can be produced synthetically. Its structure (Figure 1) includes the gentiobiose moiety (two glucose units) as well as a mandelonitrile moiety (benzyl alcohol and nitrile).^38,39^ These moieties are considered as models due to the functional groups each contains, e.g., hydroxyl and nonfused aromatic rings, that could give rise to similar spectral features as those of the higher-mass organic compounds observed in Enceladean ice grains.^23^ A solution of amygdalin in deionized Mili-Q water was prepared, at a concentration of 0.062 M. The amygdalin solution was measured twice: immediately after preparation and 5 days after preparation (during which the solution was stored at <8 °C).

**Figure 1:**
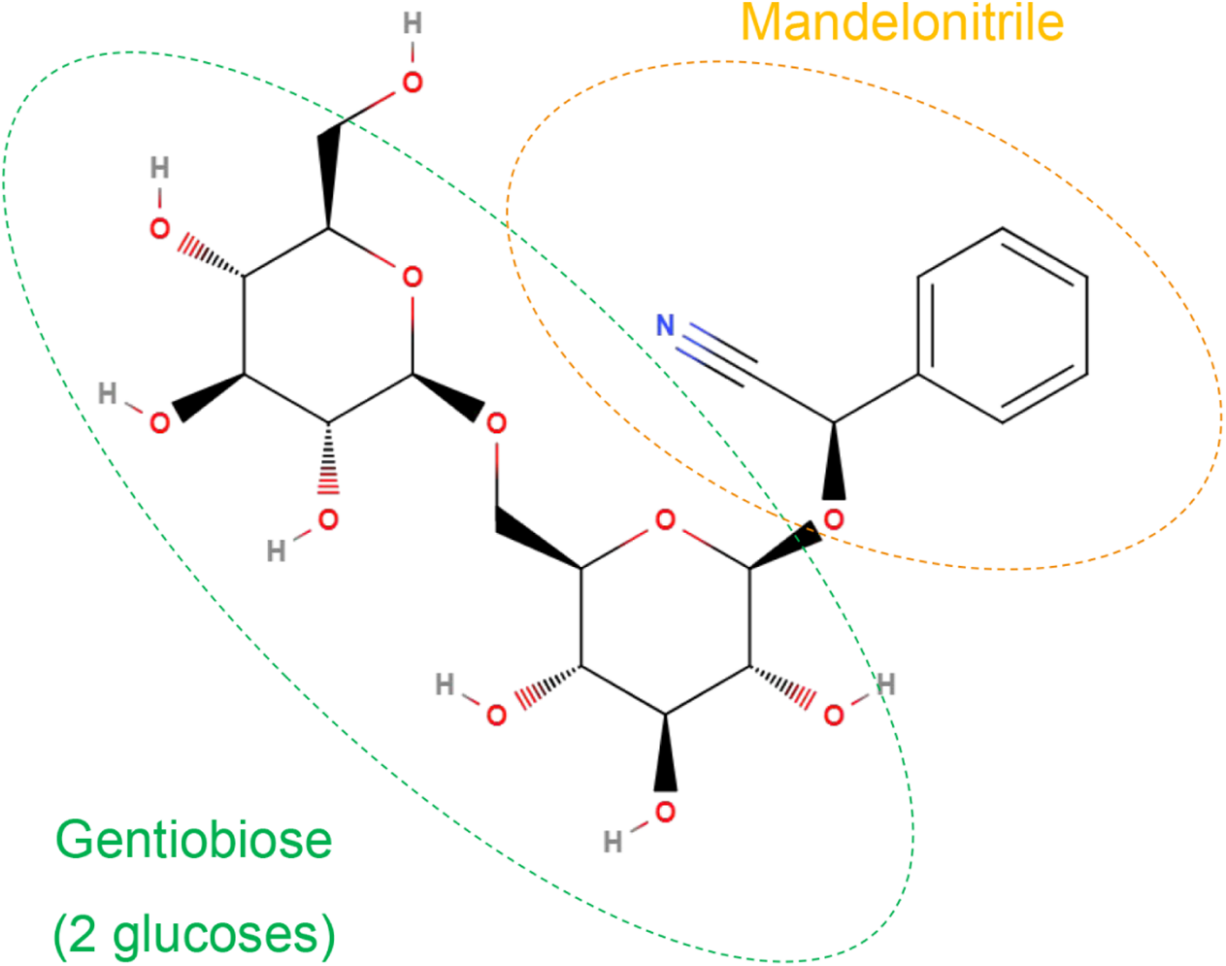
Structure of amygdalin (C_20_H_27_NO_11_) with its moieties (gentiobiose and mandelonitrile) highlighted.

### Laser-Induced Liquid Beam Ion Desorption Mass Spectrometry

Time-of-flight mass spectra of amygdalin were acquired, using the principle of delayed extraction, in cation and anion mode of the LILBID experiment. The reflectron-type time-of-flight mass spectrometer was purchased from Kaesdorf and operates at ∼10^–7^ mbar. Mass spectra are obtained with a mass resolution m/Δm of 600–800. The laser used is a pulsed infrared laser, operating with a laser energy of 5.4 J at a 2840 nm wavelength. A full description of the experimental setup can be found in the literature.^17^

The fresh solution cationic mass spectra were recorded with delay times of 6.7 and 7.5 μs, and laser intensities of 97.3% and 96.2% respectively. An anionic mass spectrum was recorded with 91.5% laser intensity and a delay time of 6.4 μs. The higher delay time of 7.5 μs was used in to facilitate the selection of higher-mass peaks in cation mode. For the five-day old solution, a spectrum with laser intensity of 80.6% and a delay time of 6.7 μs was recorded, to check for changes in spectral appearance over time (i.e., fresh versus old solution). The delay time and laser energy configurations chosen in LILBID simulate impact ionization mass spectra of ice grains recorded at approximately 4–8 km/s.^17^ All peaks in both modes correspond to singly charged species. The resulting mass spectra were recalibrated and baseline corrected, then analyzed in order to (i) determine the general fragmentation pattern resulting from the ionization process, and (ii) investigate the reactivity of amygdalin upon laser desorption.

### Nuclear Magnetic Resonance

In addition to LILBID measurements, nuclear magnetic resonance (NMR) spectroscopy measurements were carried out in order to investigate if the chemical changes observed in LILBID spectra occurred in the LILBID ionization/desorption process, or prior to it (i.e., in solution). NMR is a common analytical tool used to characterize molecular structures and study molecular interactions.^40,41^

All NMR measurements were carried out at 80 MHz on a Magritek Spinsolve 80 NMR spectrometer and the ACDLabs software was used to acquire the Fourier-transformed spectra.

A solution of amygdalin in 0.6 mL of D_2_O (99% D), approximately 0.15 M, was measured with NMR. Proton nuclear magnetic resonance (^1^H NMR) spectra were obtained immediately after solution preparation and 4 days later, to study changes in solution. Carbon-13 nuclear magnetic resonance (^13^C NMR), multiplicity-edited heteronuclear single quantum correlation (ME-HSQC ^1^H/^13^C), and heteronuclear multiple bond correlation (HMBC ^1^H/^13^C) spectra were obtained, to conclusively identify amygdalin as the sole analyte in solution.

Following this, tosylic acid (TsOH) was added to the solution (molar ratio amygdalin/TsOH = 1:0.75; 99% purity, obtained from Merck), and measured under the same conditions. TsOH is a proton donor, and was added to replicate the proton rich environment created upon matrix ionization during LILBID measurements. ^1^H NMR spectra were also acquired. The amygdalin-TsOH solution was then heated up to 80 °C for 6, 24, 72, and 144 h. ^1^H NMR, ^13^C NMR and HSQC-ME ^1^H/^13^C spectra were obtained to investigate the reactivity of amygdalin.

## Results

### LILBID Mass Spectra of Fresh Amygdalin Solution: General Spectral Characteristics

In the cation spectra, molecular fragments and their water clusters [M­(H_2_O)_n_]^+^ are located, in their protonated form, in the region *m*/*z* 18–440. Na^+^ and K^+^ ions and their water cluster series, originating from sample contamination, at low concentrations (<10^–8^ mol/L), are also observed in this region. The spectral region between *m*/*z* 458 and 608 comprises protonated molecular peaks of amygdalin and other similar-mass species as well as their sodiated adducts.

LILBID cation mass spectra, including an amplified region of interest, recorded with a delay time of 6.7 μs, as well as the anion spectra, recorded with a delay time of 6.4 μs, for the amygdalin solution (61.94 mM) can be found in the Supporting Information Figures S1, S2 and S3. The detection limit was measured for a deionized water matrix in cation mode. The base peak of the spectrum could still be clearly observed at a concentration of 0.1 mM. Peaks are identified and labeled in the Supporting Information Table S1. Similar assignments can also be found in Supporting Information Table S2 for the anion spectra of amygdalin. Commentary on the assignments can also be found in the Supporting Information.

In the cation spectral region at *m*/*z* 18–440 (Supporting Information Figure S1), where molecular fragments can be found, *m*/*z* 31 and 45 appear. CDA spectra of macromolecular organics found in Enceladus show these peaks are linked with the presence of heteroatoms in the parent molecule.^23^ For amygdalin, they correspond to fragments containing O atoms. Also featured in CDA spectra, peaks *m*/*z* 77 and 91 indicate the presence of phenyl C_6_H_5_- and benzyl C_7_H_7_- moieties in the parent molecule(s), respectively.^23^ In the present work, a peak at 77 could be assigned to a [Na+(H_2_O)_3_]^+^ cluster. However, expansion of the spectrum reveals two differentiated peaks around *m*/*z* 77. One of them is assigned to the aforementioned sodium water cluster, while the other can be tentatively assigned to the phenyl carbocation [C_6_H_5_]^+^ (Supporting Information Figure S2). The peak at *m*/*z* 91 does not coincide with any other water or sodiated cluster and can be assigned to the tropylium ion [C_7_H_7_]^+^ arising from the benzyl group in the mandelonitrile moiety (see Figure 1).

Further CDA spectral features characteristic of macromolecular organics such as the mass intervals between peaks of 12u or 13u (where u is the unified atomic mass unit) and the abundance of intense nonwater peaks below *m*/*z* 80^23^, do not appear in the cation spectra of amygdalin.

The anion spectra confirm the presence of molecular peaks of amygdalin and other similar-mass species, shown in Figure S3 as deprotonated or anionic molecular peaks.

### LILBID Mass Spectra of Fresh Amygdalin Solution: Reactivity

Given the detection of multiple molecular peaks above *m*/*z* 458, a spectrum at a higher delay time was acquired to facilitate the selection of higher-mass peaks. The LILBID cation mass spectrum of amygdalin recorded with a delay time of 7.5 μs (Figure 2) reveals several protonated molecular peaks, including amygdalin. The base peak of the spectrum can be observed at *m*/*z* 475 as well as the protonated molecular peak of amygdalin at *m*/*z* 458. Notably, the difference in intensity between the base peak and protonated molecular peak of amygdalin hints at the high abundance of the species detected at *m*/*z* 475. The identity of this feature cannot be assigned to a water cluster of protonated amygdalin nor any sodiated adduct, and is identified as protonated α-hydroxy-amygdalone (Figure 3). The unexpected appearance of species other than amygdalin in the spectra requires further analysis in order to elucidate their synthesis. Intermediate species in the formation of α-hydroxy-amygdalone present protonated molecular peaks in the spectrum shown in Figure 2:

**Figure 2:**
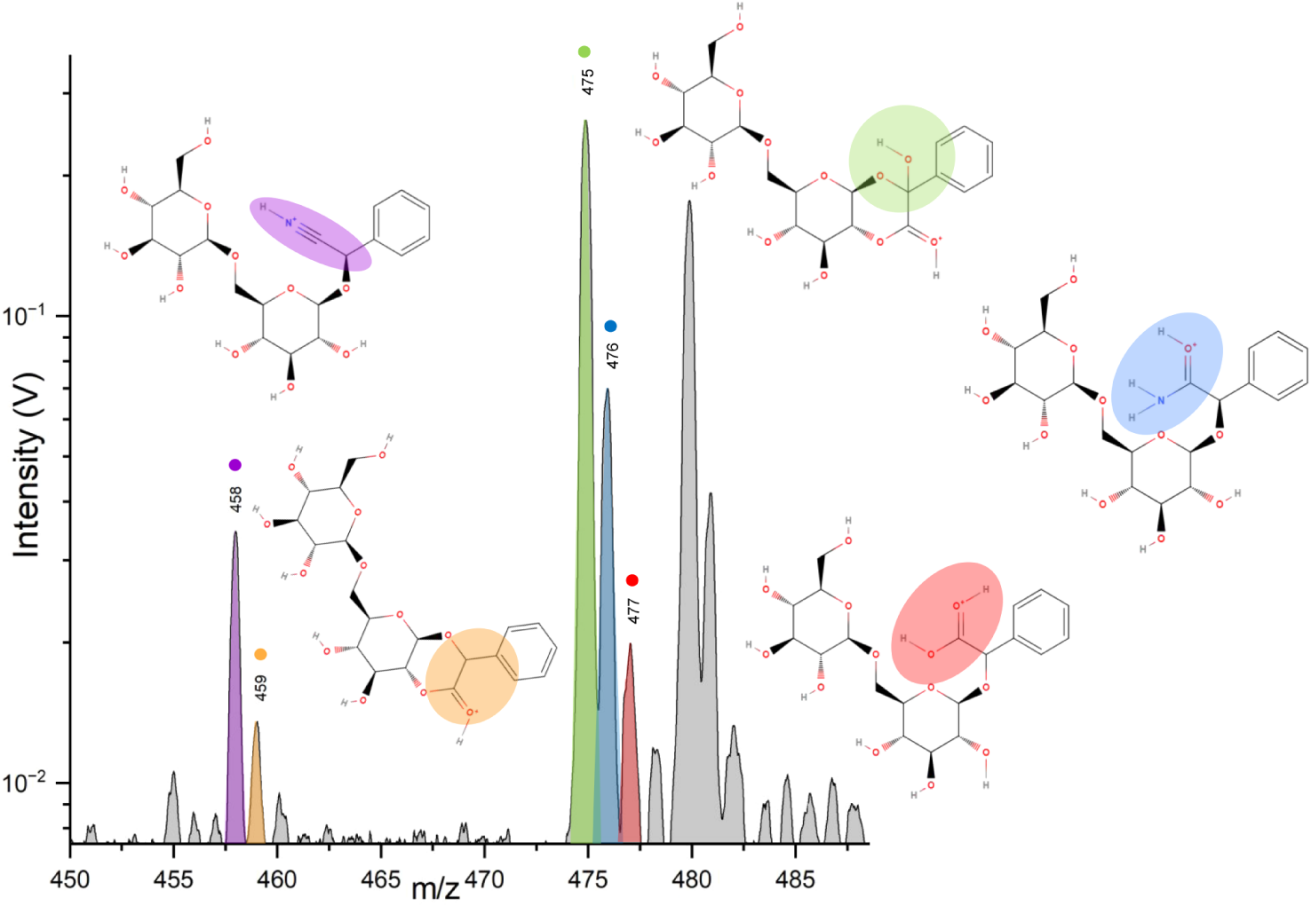
Section from the LILBID cation mass spectra of a fresh amygdalin solution in deionized water, recorded at a delay time of 7.5 μs, showing *m*/*z* values between 450 and ∼478. Protonated molecular peaks of amygdalin (*m*/*z* 458), amygdalone (*m*/*z* 459), α-hydroxy-amygdalone (*m*/*z* 475), amygdalin amide (*m*/*z* 476) and amygdalinic acid (*m*/*z* 477), are labeled and assigned to their corresponding molecular structure. Molecular structures include highlighted areas indicating distinct functional groups of each molecule. Their related formation pathways are shown in Figure 4. The *m*/*z* of the peaks are rounded to integer numbers.

**Figure 3:**
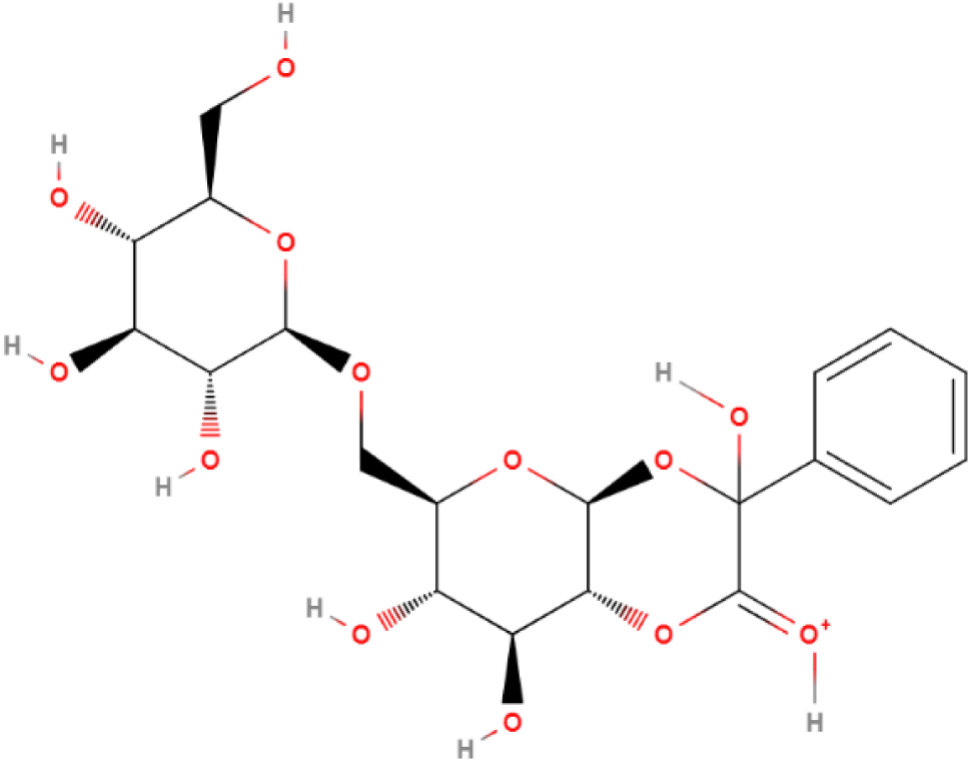
Structure of protonated α-hydroxy-amygdalone, observed at *m*/*z* 475 in Figure 2. The stereocenter in α to the carbonyl group is not defined.

•*m*/*z* 476, assigned to the protonated amygdalin amide intermediate,

•*m*/*z* 459, assigned to the protonated lactone form of amygdalin (i.e., amygdalone),

*m*/*z* 477, assigned to protonated amygdalinic acid. A water cluster of the protonated molecular peak of amygdalin could also contribute to the peak observed at *m*/*z* 476. Similarly, a water cluster of the lactone form of amygdalin (*m*/*z* 459) could contribute to the peak observed at *m*/*z* 477. These contributions, however, would not be significant. A more detailed explanation on water cluster contributions to the molecular peaks detected can be found in the Supporting Information, section 1.

#### Hydrolysis of the Nitrile Group

In conventional organic chemistry literature,^42^ the hydrolysis of nitriles to yield carboxylic acids is catalyzed by acids or bases and involves an amide intermediate. Initially, the amide is formed, but since amides are also hydrolyzed under acidic or basic conditions, the carboxylic acid is readily obtained. In this case, however, the amide intermediate is sufficiently stable to be observed in its protonated form (Figure 2). The product of nitrile hydrolysis, amygdalinic acid, can also be observed.

#### Intramolecular Esterification

Regardless of the extent of hydrolysis of the nitrile group, both amide and carboxylic acid derivatives undergo further transformation, namely intramolecular esterification with a hydroxyl group present in the nearest sugar from the gentiobiose moiety. The intramolecular esterification produces the lactone amygdalone. Its protonated form (*m*/*z* 459) is depicted in Figures 2 and 4. It should be noted that, in solution, esterification reactions also require acid-catalysis (protonation) in order to proceed.

**Figure 4:**
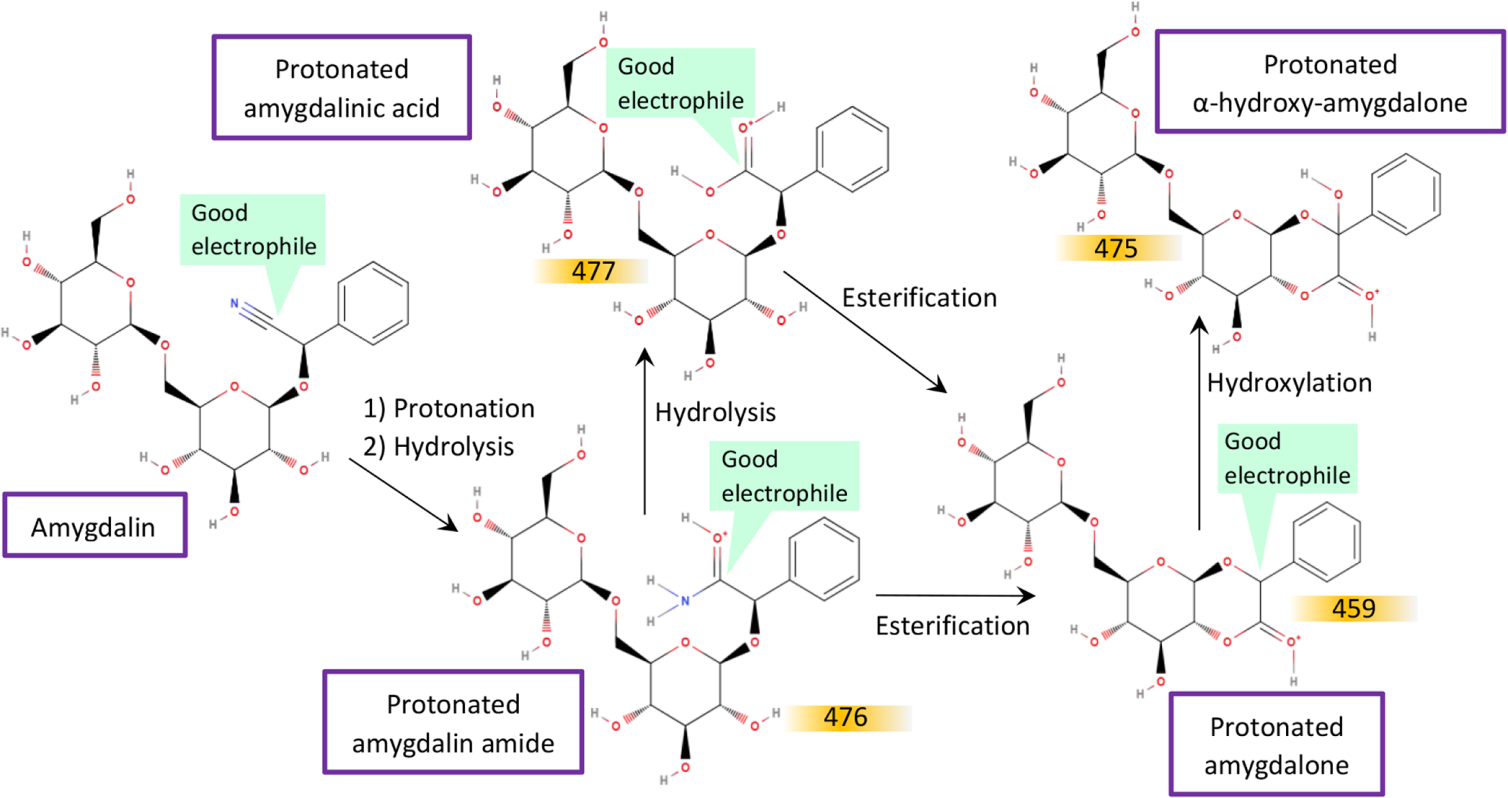
Reaction scheme for the obtention of α-hydroxy-amygdalone from amygdalin, in LILBID. The reaction entails hydrolysis of the nitrile group in amygdalin, subsequent intramolecular esterification and, finally, hydroxylation to give the α-hydroxy-derivative. All molecules depicted are identified in their protonated form in the mass spectra in Figure 2, at the *m*/*z* labeled in orange. Centers of reactivity in each molecule are indicated in green.

#### α-Hydroxylation

Amygdalone can incorporate a hydroxyl group (from the H_2_O ionized matrix) in the α position to the carbonyl group, during the last reaction step described in Figure 4, leading to α-hydroxyamygdalone as the final product. In solution, the α carbon must be activated—increase its electrophilicity—in order for a hydroxide ion to attack this position. One method of activation is acid-catalyzed tautomerization of the carbonyl moiety, leading to an enol.

Many compounds containing a carbonyl moiety present tautomerism, provided that a proton in the α carbon is present.^42^ Tautomerism is a special case of structural isomerism, in which the isomers (tautomers) are interconvertible proton transfer between atoms. In the case of keto–enolic tautomerism, relevant for compounds with carbonyl moieties, a proton is removed from the α carbon and added to the oxygen in the carbonyl (i.e., H–C_α_–C = O ⇌ C_α_=C–O–H), with the rearrangement of the double bond. Amygdalone meets the structural requirements to undergo keto–enol tautomerism, as depicted in Figure 5.

**Figure 5:**
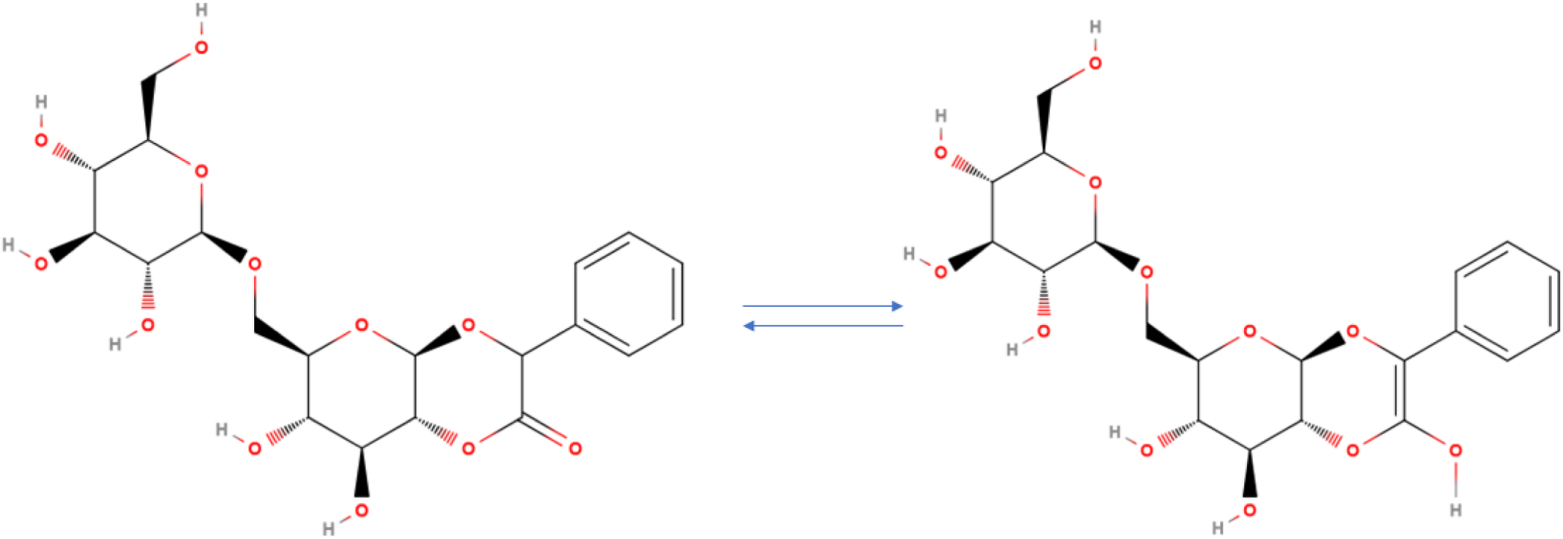
Amygdalone keto-enol tautomerism scheme, where a proton in α position (keto form, on the left) is removed and a proton is added to the oxygen from the carbonyl group (enol form, on the right).

Tautomers exist in an equilibrium (Figure 5) where the keto tautomer is usually predominant. Protonation can, however, drive the tautomeric equilibrium toward the enol form, stabilizing it via conjugation of the double bond. The lactone enol in amygdalone presents conjugation with the adjacent arene moiety, potentially increasing its stability. The proton-rich environment created during matrix ionization in LILBID would also stabilize the enol tautomer.

The fact that α-hydroxy-amygdalone is observed upon measurement with LILBID indicates the feasibility of incorporating a hydroxyl group onto the lactone’s α carbon. Conventionally, the carbon in α position to a carbonyl/carboxyl group acts as a nucleophile due to its innate polarity.^43^ This prevents the addition of a hydroxide anion (also nucleophilic) to that position, as the polarities of the reacting groups are not compatible. One explanation for this counterintuitive reaction product involves umpolung of the α carbon. The term umpolung refers to the inversion of the natural polarity of a given atom in an organic molecule.^44^ For amygdalone, protonation of its stable enol form leads to the umpolung of the α carbon (Supporting Information S4), causing it to behave as an electrophile, which is then susceptible to nucleophilic attacks by water or hydroxyl ions. The α-hydroxy-derivative is produced in this case, with an undefined stereochemistry of its α carbon, as indicated by Figure 3.

Based on these deductions, the described reaction must occur either in solution prior to measurement or upon laser irradiation with LILBID.

From the last reaction step, the presence of a lactone hints at the reaction proceeding in the gas phase. Lactone enols, as present in amygdalone (Figure 5), have been shown to be stable in the gas phase but unstable in solution,^42^ although this is not a unique interpretation. While it is clear that amygdalin can react to form such products, further analysis is required to understand when the observed reaction is taking place (i.e., in solution or during matrix ionization).

The LILBID anion mass spectrum of amygdalin recorded with a delay time of 6.4 μs shows the presence of amygdalin and its reaction products, (the same set present in cation mass spectra), as anionic and deprotonated molecular peaks (Supporting Information Figure S3). Further discussion over these molecular peaks can be found in Supporting Information section 1.

### LILBID Mass Spectra of Five-Day-Old Amygdalin Solution

To investigate whether this reaction occurs prior to or during measurement with LILBID, the same solution was measured again 5 days after the first set of measurements, using the same experimental settings. The results (Supporting Information Figure S5) show no appreciable changes to the spectral appearance when compared to the fresh samples (Supporting Information Figure S1). This lack of changes indicates an equilibrium in solution, but does not give definitive proof on whether the reactions have occurred partially, completely, or not at all—prior to measurement. Some reactions described in Section 2 of this work^42^ establish equilibrium in solution, where both reactants and products coexist.

Since LILBID mass spectral analysis cannot provide definitive proof as to the timing of the reaction, NMR measurements of amygdalin in deuterated water were carried out in order to test its stability in solution and the timing of the reaction observed.

### Amygdalin NMR Measurements

#### Fresh and Four-Day-Old Amygdalin Solution: Proton Spectrum

In order to understand exactly when the reactivity described in Figure 4 is happening, (in solution prior to measurement or as a result of ionization of the water matrix), an amygdalin solution in D_2_O was measured using NMR spectrometry. ^1^H NMR was performed to confirm the presence of the analyte amygdalin and test its stability in the aqueous medium. ^1^H NMR spectra were obtained both immediately after preparation and after 4 days undisturbed. There were no significant changes between the spectra obtained directly after preparation of the solution and 4 days after preparation. Thus, the spectrum presented in Figure 6 corresponds to fresh amygdalin solution but is also representative of the four-day-old solution spectrum. The NMR spectrum recorded 4 days after solution preparation can be found in Supporting Information Figure S6. NMR spectral analysis was carried out to identify the analyte(s) present in solution.

**Figure 6:**
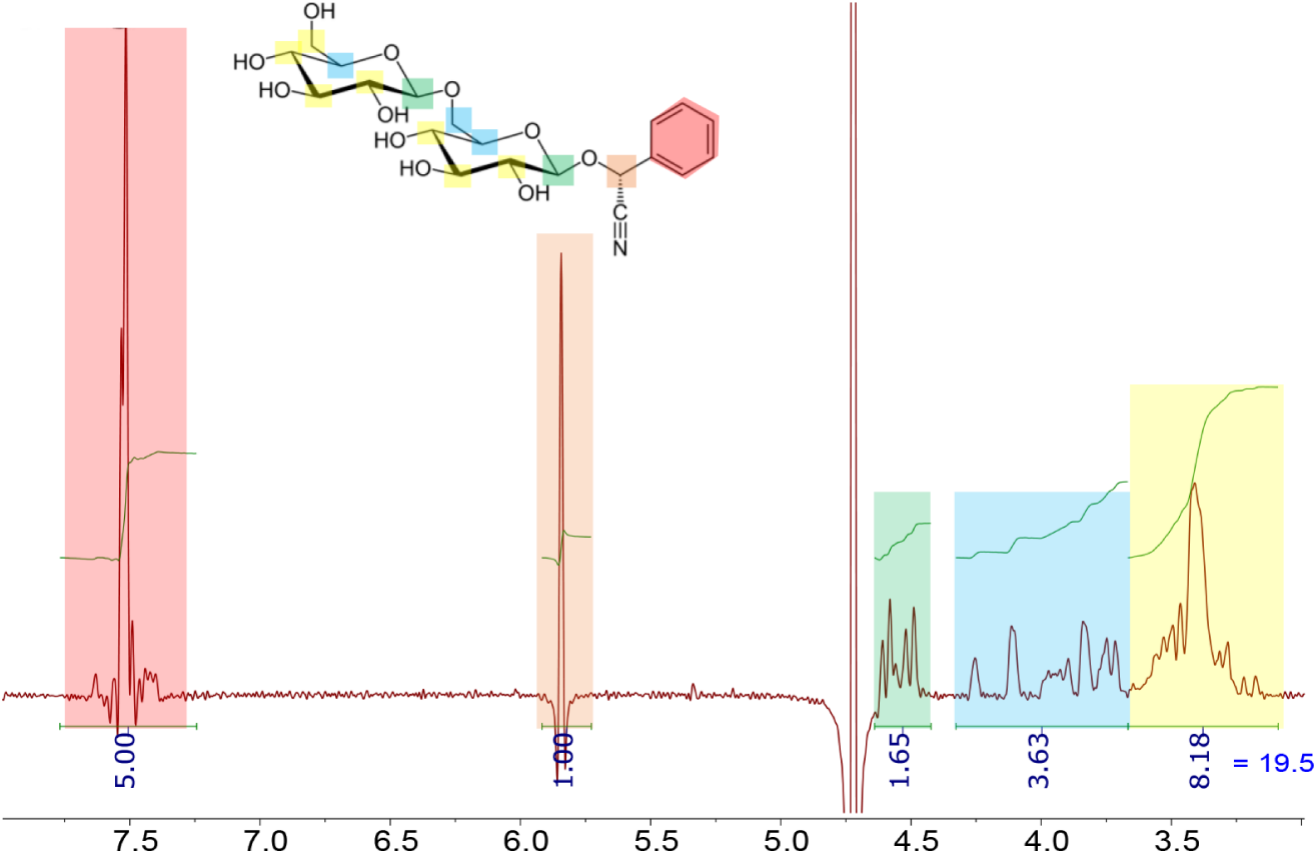
^1^H NMR (D_2_O, 80 MHz) carbon-decoupled spectrum of fresh amygdalin solution. The *x* axis represents the chemical shifts in ppm. The integrated area under each peak is proportional to the number of nuclei (^1^H) with the same chemical shift. Peak integrals are shown in green and integral numbers in blue. The solvent signal can be seen at approximately 4.75 ppm.

Downfield from the solvent peak at 4.75 ppm,^45^ two signals are present: a singlet at 5.84 ppm and a group of peaks around 7.51 ppm. The latter is assigned to the five aromatic protons in the phenyl moiety, as indicated by the integral number in blue (Figure 6). Their chemical shift is also in accordance with this assignment.^45^ The signal at 5.84 ppm is assigned to the proton in the benzylic position. This position is surrounded by various electron-withdrawing functional groups, e.g., phenyl, nitrile and alkoxy, whose cumulative effect results in an increased deshielding of its aliphatic proton.^45^

Upfield from the D_2_O signal, three groups of peaks are observed. The integrated area of the peaks around 4.5 ppm corresponds to two protons, with similar chemical environments in the molecule. They are tentatively assigned to the acetal protons, because of the double deshielding effect of the surrounding alkoxy groups.^45^

The last two groups of signals appear around 4 and 3.3 ppm, upfield from the acetal protons but still significantly deshielded. Integrated areas, corresponding to four protons for the former and eight protons for the latter, point to two distinct chemical environments. The four protons at higher frequencies can be assigned to the methylene groups contiguous to the acetal moieties. The eight protons at lower frequencies correspond to the methylene and methine groups contiguous to the hydroxy moieties present in the structure of amygdalin.^45^ Protons belonging to hydroxyl groups would not appear in the spectra due to rapid exchange with the deuterated aqueous medium.

The structures of amygdalin and its amide, carboxylic acid, lactone and hydroxy-lactone derivatives are fairly similar. Thus, molecular structure determination should focus on characteristic spectral features arising from structural differences. The presence of a proton at 5.84 ppm cannot be explained if α-hydroxy-amygdalone, identified in the LILBID spectral analysis, is taken as the analyte in solution: there exists no proton position in the structure that would justify the appearance of a singlet at such high frequency.

#### Fresh and Four-Day-Old Amygdalin Solution: Carbon Spectrum

In order to differentiate amygdalin from other possible derivatives observed in LILBID spectra, ^13^C NMR spectral analysis was performed in the fresh amygdalin solution.

The ^13^C NMR spectrum facilitates the unequivocal identification of the analyte as amygdalin. In Figure 7, the absence of signals in the 170–190 ppm region of the ^13^C NMR spectrum -where signals from carboxylic compounds and derivates would appear—indicates that no transformation of the nitrile group into any carboxylic acid derivative has taken place. Therefore, this indicates that the reactions described in Figure 4 take place during matrix ionization in LILBID measurements, most likely on the order of nanosecond time scales.^46–48^

**Figure 7:**
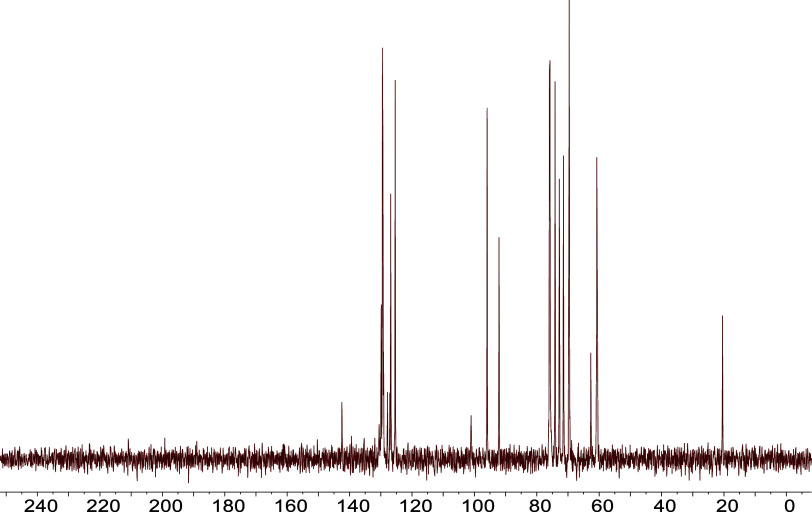
^13^C NMR (D_2_O, 20 MHz) proton-decoupled spectrum of a fresh amygdalin solution. The *x* axis represents the chemical shifts in ppm. The absence of signals in the 170–190 ppm region indicates the only analyte present in solution is amygdalin.

Further 2D NMR measurements were carried out—ME-HSQC ^1^H/^13^C and HMBC ^1^H/^13^C—to confirm the assignments made in the 1D NMR experiments. The 2D measurement results are shown and discussed in Supporting Information Figures S15 and S16.

Additional NMR experiments measured amygdalin in a heated acidic solution in the presence of tosylic acid. The results show that the reaction pathway in the presence of an acid is distinct from the reaction pathway deduced from LILBID results. A detailed discussion of the results obtained from these experiments can be found in the Supporting Information Figures S7–S14 in addition to Figures S17 and S18). These results highlight the unique reactivity of amygdalin during LILBID measurements, triggered by ionization of the water matrix. Supporting Information Figure S19 shows the structure of amygdalin, indicating the chemical shifts of carbons and protons relevant for structure elucidation, obtained in D_2_O.

## Discussion

### Comparison of Amygdalin’s Spectral Signature with Organic Species Observed with CDA in Ice Grains from Enceladus

Analysis of LILBID spectra of amygdalin show the presence of peaks assigned to some of the moieties discussed in the work related to low- and high-mass organic compounds observed in CDA spectra of ice grains from Enceladus.^23,25,26^ Peaks at *m*/*z* 31 and 45 are assigned here to fragments containing O whereas peaks at *m*/*z* 77 and 91 indicate the presence of the phenyl and benzyl moieties, respectively.^23,25^ Amygdalin presents only a benzyl functional group, not a phenyl functional group, and cannot be the parent molecule giving rise to the peak at *m*/*z* 77.

Peak analysis, however, shows the presence of molecules other than amygdalin, obtained as a consequence of the reactivity of the analyte induced by proton-rich matrix environment upon measurement. One of the products obtained from this reaction, α-hydroxy-amygdalone (see Figure 4), could yield the observed peak at *m*/*z* 77, given its fully substituted benzylic position. This means, upon fragmentation, the molecular structure does not allow for the incorporation of a C–H group into the ring, rather favoring the formation of phenyl cations (*m*/*z* 77).

Further spectral features discussed in previous works^23,25^ were not present in the spectra of amygdalin. Other high-mass compounds should be studied in future to identify the organic structure giving rise to the spectral fingerprints described in these works, taking into account the positive spectral matches described here.

### Reactivity upon Laser-Induced Matrix Desorption

LILBID measurements of the analyte amygdalin resulted in the detection of chemical reactivity: (i) the hydrolysis of the nitrile group of amygdalin, followed by (ii) subsequent intramolecular esterification of the amide/carboxylic acid intermediate with the hydroxyl group of the adjacent sugar and, finally, (iii) α-hydroxylation of the lactone resulting in α-hydroxy-amygdalone. Further NMR measurements were performed in order to discard the possibility of reactivity occurring in solution prior to measurement with LILBID. NMR results for the fresh solution establish that the amygdalin in solution does not react or decompose into other products and is stable in solution for at least up to 4 days. This is in good agreement with results from LILBID, where no significant changes were observed between spectra collected from fresh and five-day-old solutions. Even in the presence of strong acids that could trigger the observed reactivity, no reaction takes place without heating of the solution. Moreover, upon heating, no peaks appear in the 170–190 ppm region in the carbon-13 NMR spectra, where the carboxylic derivatives observed in LILBID would appear. This indicates that hydrolysis of the nitrile group of the mandelonitrile moiety in amygdalin does not take place in the condensed phase, rather amygdalin decomposes into its constituent moieties. This is an important deviance from the reactivity observed in LILBID. We therefore conclude, that reactivity observed in LILBID falls into the newly coined category of protonation-induced chemical transformations (PICTs) facilitated by matrix decomposition. This term includes chemical transformations of analytes induced by the particular environment to which they are subjected upon measurement with LILBID. The occurrence of certain PICTs during IR laser-induced reactions under supercritical conditions has been recorded previously.^46^ The distinct PICTs discussed in this work broaden the inventory of reactions possible under such conditions.

The PICTs observed for amygdalin centers on the nitrile group specifically and develops due to protonation and interaction with water molecules in its immediate vicinity. The reaction steps take place either between the organic molecule and the matrix (e.g., hydrolysis, hydroxylation, protonation), or are entirely intramolecular (e.g., esterification). The chemical reactions observed must take place likely on the order of nanosecond time scales.^46–48^ For intramolecular reactions, this is a reasonable timespan similar to the ones described in other works.^49–51^ Intermolecular reactions require the close proximity of water molecules around the analyte during matrix ionization.

Reactivity of the nitrile group, triggered by protonation, seems to be independent of the other moieties (glucose) present in the compound. This hints at other compounds featuring nitrile groups also being susceptible to reactivity upon water matrix ionization. Functional groups such as carboxylic acid and amide can also experience reactivity (cyclization/esterification) due to a highly protonated environment. The observed reactivity involves additional functionalities (alcohol groups) and might be more restricted or absent in other molecules lacking them. Other cyclization reactions that, in conventional organic chemistry literature, also benefit from protonation (e.g., Diels-Adler reactions^52^) should also be explored. Reduction reactions of aromatic compounds due to intense protonation have been detected under similar conditions.^46–48^

Species with relatively labile protons, like the carbonyl group featured in amygdalone in this work, can also present reactivity upon protonation. Tautomeric compounds are also susceptible to such protonation-induced reactivity, incorporating new functionalities in the α position. The addition of heteroatomic functional groups (such as −OH) in the analyte as a result of protonation and hydroxylation, is a phenomenon that should be further studied in future work.

### Implications for LILBID Work and Impact Ionization Mass Spectrometry

One example of a spectral feature that could represent an unconfirmed case of reactivity induced by water matrix ionization can be found in the work of Khawaja et al.^31^ They studied the decomposition of triglycine peptide under hydrothermal conditions, and its spectral appearance pre- and post- hydrothermal processing with LILBID. They assigned a spectral feature to diketopiperazine, a common decomposition product of short peptides^53^ formed through intramolecular cyclization and found in the spectra of both processed and unprocessed triglycine. Although more detailed analyses were not performed, the intramolecular cyclization required for peptides to form diketopiperazine is not unlike the one described in this work (intramolecular esterification and formation of an amide). This could hint at some degree of reactivity due to matrix decomposition and protonation of the analyte, and merits further investigation in light of the current work.

Dust accelerators on Earth can produce impact ionization mass spectra of large molecules in water-poor dust, in contrast to ice grains from Enceladus and LILBID measurements, providing reference for spaceborne impact ionization instruments such as the DESTINY+ Dust Analyzer.^54^ Recorded impact ionization mass spectra of anthracene^55^ show a series of peaks separated by intervals of 12 u, attributed to the recombination of neutral carbon atoms or CH radicals onto the protonated molecular ion. In contrast, the reactivity we observe in amygdalin is different in nature and involves intramolecular as well as intermolecular reactivity with adjacent water molecules, which are not reported in the anthracene experiments. The difference in reactivity between the two experiments is linked with the presence/absence of water. The reactivity of amygdalin is induced due to the decomposition of the water matrix in which the analyte is dissolved. The clustering of water molecules onto protonated molecules is thus likely to be competitive with the addition of C­(H) fragments in water-rich samples, if the latter does occur at all. Indeed, no reactivity between two molecules of the analyte is reported in this work.

The reactivity observed for anthracene is characteristic to an impact velocity between 10–16 km/s.^55^ Thus, anthracene shows a form of impact cloud chemistry dependent upon the availability of free carbon atoms, which may not be liberated at lower impact speeds. Many sampling conditions experienced by SUDA-type mass spectrometers analyzing ice grains may not facilitate PICTs like the one described here. For instance, the CDA data obtained during the highest velocity flybys of Enceladus by Cassini (∼17.7 km/s) may be more comparable to data acquired by other mass spectrometric techniques, such as electron ionization,^26^ as the formation of water clusters—and indeed formative chemistry in general—is inhibited at such speeds. This, in turn, could hinder proton mobility in the gas phase aggregates and diminish reactivity.

Nevertheless, LILBID remains a vital laboratory analogue technique to replicate the mass spectra of ice grains obtained by spaceborne mass spectrometers, such as SUDA, at a broad range of velocity regimes.^28,31–34^ Despite clear differences between the two methods (such as the aggregation state of the sample prior to measurement, i.e., ice particles vs water beam), impact ionization mass spectrometers could also trigger reactivity of targeted analytes embedded in ice grains upon measurement.

LILBID is characterized by at least three components that impact the outcome and features of the desorption and ultimately fragmentation: (1) protonation and deprotonation in a very hot water phase, (2) plasma ionization, where real ionization can occur, and (3) clustering and charge recombination. High temperature, ≈1750 K, chemical reactions in the supercritical water phase (PICTs), when present, represent a fourth component.^46^ Thus, it is always a mixture of the three (four if PICTs take place) processes that occur during LILBID. As the laser energy changes, so does the contribution of each process, which in turn results in distinct spectral features.

In space, the variability in spectral appearance due to different velocity regimes would indicate the ice particle impact also has several (mechanistic) components. It is not just a cold plasma that is well-simulated by LILBID but also the energetic conditions imparted by the laser. This is also the reason why LILBID conditions (energies) that mimic the ice impacts at certain kinetic energies can be found. Thus, under certain velocity regimes, PICTs akin to those observed in this work are relevant to both scenarios, as both impact ionization and laser dispersion enable protonation of analytes in the postionization cloud. The extent of gas phase reactivity and its dependence on the impact speed of ice grains onto spaceborne mass spectrometers has not yet been explored and merits further work.

This fact must also be taken into consideration for the analysis of data from past, ongoing, and future space missions. Furthermore, the recent emergence of electrostatic accelerators that allow direct replication of the impact ionization process of ice grains onto space detectors^56,57^ should be used to assess the effect of the matrix aggregation state on the reactivity of compounds contained therein.

## Conclusion

We measured the model high-mass compound amygdalin with LILBID mass spectrometry to study and contrast its spectral features to those of high-mass complex organics detected by CDA in Enceladean ice grains. Only a few spectral features of amygdalin matched that of the observed high-mass organics.

Unexpected protonation-induced chemical transformation (PICT) of amygdalin due to matrix ionization was observed and verified by NMR measurements. PICTs during IR laser-induced supercritical phase reactions have been identified previously.^46^ However, to the best of our knowledge, the consecutive PICTs detected in this work constitute distinct reactions, identified in the gas phase upon measurement of LILBID. The nature of the reactivity observed, triggered by water matrix disintegration and consequent protonation of the analyte, may also be relevant for spaceborne impact ionization mass spectrometers as well, depending upon impact speeds.

Past and ongoing space missions, such as Cassini Huygens and Europa Clipper, study the habitability and astrobiological potential of icy ocean worlds. Thus, it is of particular importance to understand the extent of PICTs in astrobiologically relevant molecules (e.g., peptides) following ice/water matrix disintegration (i.e., measurement with both LILBID or impact ionization of ice grains). Future work should study a suit of molecules containing the same target moiety, but varying structures with different accompanying functional groups. This will help derive general rules of PICTs for organic compounds. The extent of the reactivity seen for functional groups such as nitrile, carboxylic acid, amide and tautomeric compounds, should also be further studied on ice grain accelerator experiments, to understand the effect of the aggregation state of the matrix on reactivity. PICTs should be regarded as a factor that can alter the appearance of parent molecules in impact ionization spectra obtained from ice grains in space. Similarly, parent molecules can be transformed or modified by other processes such as hydrothermal processing or irradiation, concomitant to icy ocean worlds. Further work into the elucidation of data obtained from spaceborne impact ionization instruments would benefit from a holistic view of the possible effects derived from all these processes.

## Supplementary Material



## Notes

LILBID cation and anion-labeled spectra and tables, charge distribution calculations for amygdalin, 1D and 2D NMR spectra of amygdalin and glucose, and NMR assignation of amygdalin (PDF)
