# Supplementary material for: Protonation-Induced Chemical Transformations in Mass Spectrometry: Implications for Detecting Complex Organics on Icy Moons
Source: ACS Earth Space Chem. 2026 Feb 19;10(3):765–76. doi: 10.1021/acsearthspacechem.5c00363 (PMC13007013; doi:10.1021/acsearthspacechem.5c00363)
Supplement: Supplementary file 1 [file sp5c00363_si_001.pdf]

Protonation-induced chemical transformations on mass spectrometry implications for detecting complex organics at icy moons.

Lucía Hortal Sánchez[1], Maryse Napoleoni[1], Ernesto Brunet[2], Fabian Klenner[3,4], Thomas R. O'Sullivan[1], Mirandah Ackley[1], Gregoire Danger[5], Bernd Abel[6,7], Nozair Khawaja[1], Frank Postberg[1].*

[1] Institut für Geologische Wissenschaften, Freie Universität Berlin, Berlin, 12249, Germany;

[2] Department of Organic Chemistry, Universidad Autónoma de Madrid, Madrid, 28049, Spain;

[3] Department of Earth and Space Sciences, University of Washington, Seattle, USA;

[4] Department of Earth and Planetary Sciences, University of California, Riverside, 92521, USA

[5] Aix Marseille Univ, CNRS, Institut Origines, PIIM, Marseille, 13013, France;

[6] Institute of Chemical Technology, University Leipzig, Leipzig, 04103, Germany;

[7] Department Space Chemistry and Technology, J. Heyrovsky Institute of Physical Chemistry, Czech Academy of Sciences, Praha, 182 23, Czech Republic.

*corresponding author: lucia.hortal@fu-berlin.de

Supporting Information

1. Fresh amygdalin solution cation and anion LILBID spectra and assignation

Cation mode spectra

Standard patterns of water clustering for cation spectra are observed with the formula $[H_3O+(H_2O)_n]^+$. Organic-related peaks can also form water clusters at increasing masses with intervals of 18 u [1, 2]. Sodiated adducts $[M+Na]^+$ can also experience clustering of water molecules, presenting the same interval of increase in mass. Analysis of the most important fragments, water clusters and sodiated adducts was carried out in the spectra recorded at 6.7 µs.

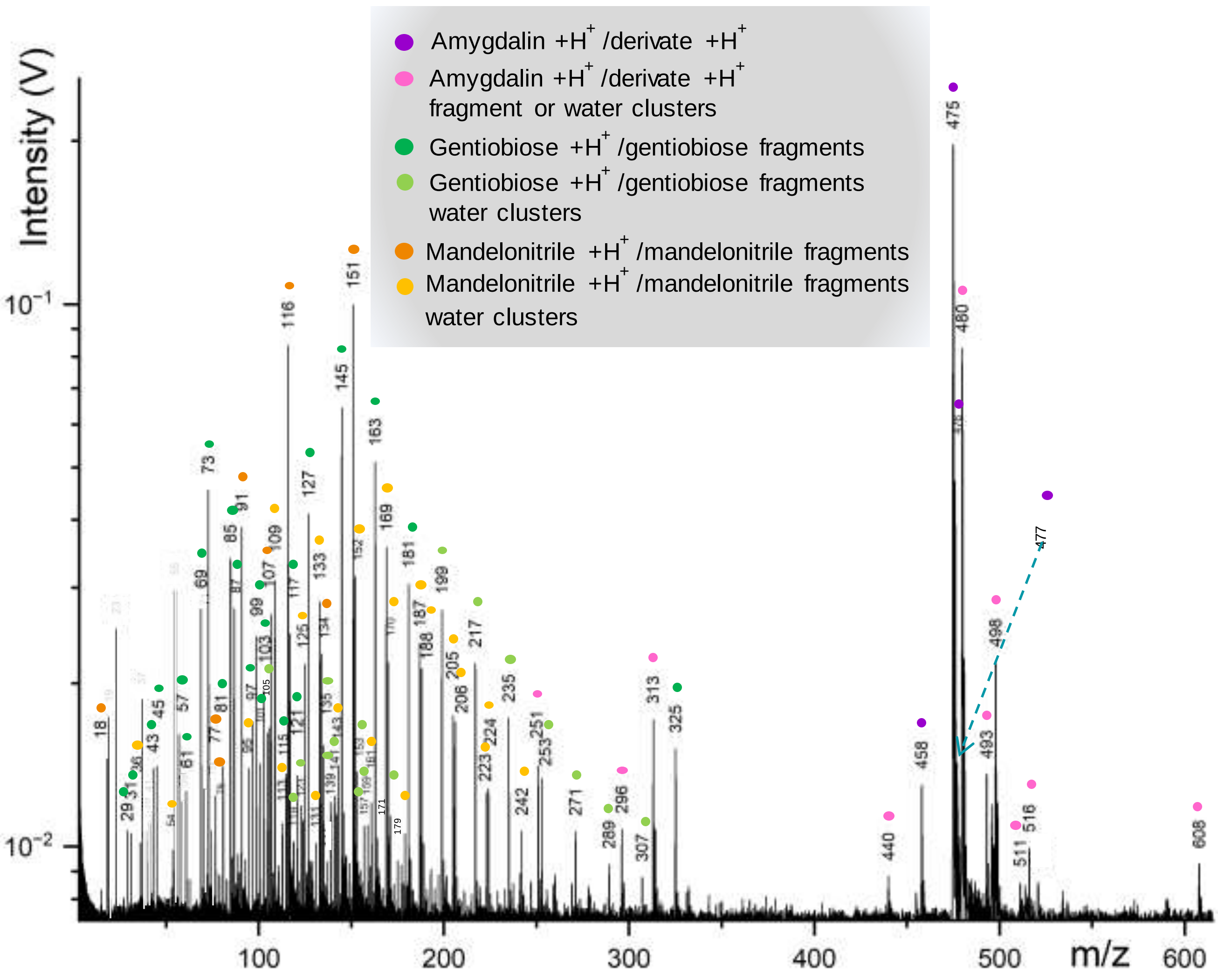

Figure S1: Baseline corrected LILBID cation mass spectra of a fresh amygdalin solution in deionized water, recorded at a delay time of 6.7 µs. Assignation of the labelled peaks to proposed fragments can be found in **Table S1**. Pure water, Na^+ and K^+ water cluster peak series are partially shaded in order to facilitate spectra analysis.

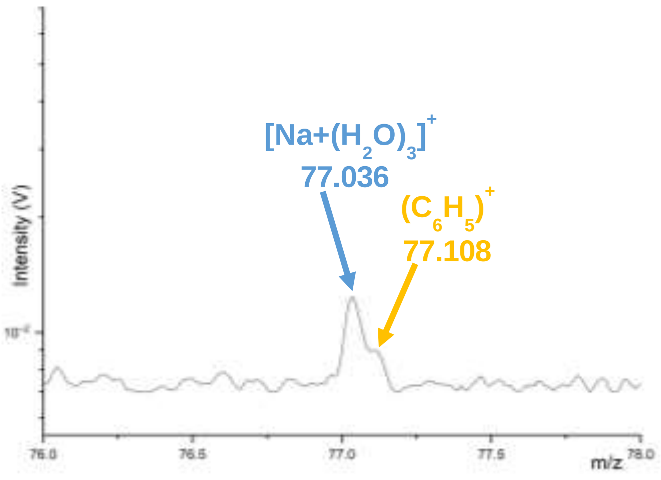

Figure S2: Expansion of the spectral region between m/z 77 and 79 of the cation spectra in **Figure S1**, showing two peaks around mass 77 u.

In the higher-mass range of the spectra in Figure S1, m/z 400-550, where the molecular peaks appear, the contribution of water clusters to the assigned molecular peaks can be assessed. Water clusters of the base peak, m/z 475, occur at m/z 493 and 511. Their intensity is very significantly below the intensity of the base peak. If we translate this relationship between the intensity of the molecular peak and the intensity of its water clusters to other molecular peaks, it can be deduced that water clusters of m/z 458 or m/z 459 would not contribute significantly to the (more intense) peaks at m/z 476 and m/z 477, respectively.

The minimum resolution (R_{min}) needed to distinguish the two peaks shown in **Figure S2** can be defined as $R_{min} = \frac{m}{\Delta m}$ where m_1 = 77.0363 and m_2 = 77.1086. R_{min} = 1065.51.

The resolving power at this mass is defined as $resolving\ power = \frac{m}{FWHM}$ with FWHM calculated for m_1. The resolving power has a value of 1213.76. Thus, it is possible to treat the peaks in **Figure S2** as separate and differentiated peaks (Resolving power > R_{min}). Guided by the theoretical masses expected of each stoichiometric formula, the peak on the left is tentatively assigned to

the $[Na+(H_2O)_3]^+$ sodium water cluster and the peak on the right to a $[C_6H_5]^+$ phenyl carbocation fragment.

Anion mode spectra

Anion mode ToF mass spectra were recorded to complement the cation mode spectral analysis. The spectra recorded with a delay time of 6.9 µs can be found in **Figure S3** and partial peak assignation can be found in **Table S2**. The abbreviation used in **Table S2** amygdalin is the same as used for **Table S1**. The observed spectral features also show the presence of amygdalin and its reaction products identified in cation mode. Standard patterns of water clustering are observed, with the formula $[OH+(H_2O)_n]^-$. Organic-related peaks can also form water clusters at increasing masses with intervals of 18 u [1,2].

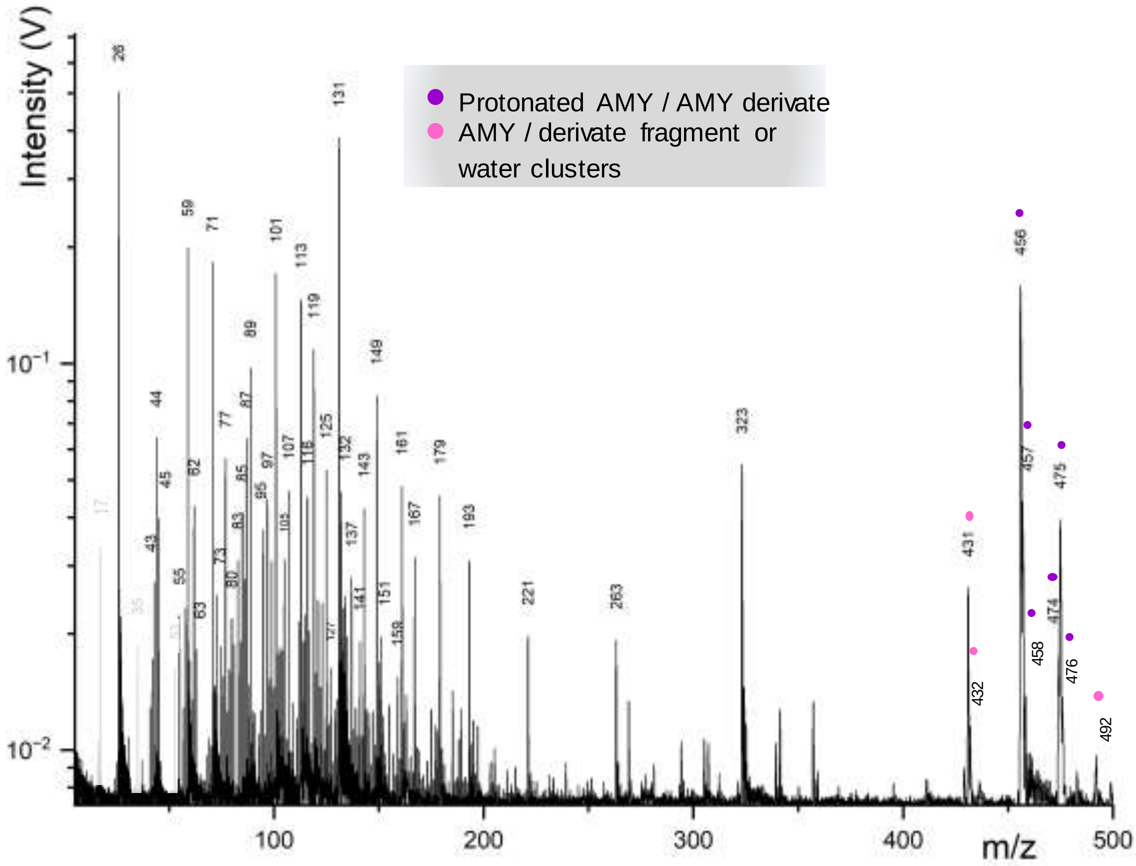

Figure S3: LILBID anion mass spectra of a fresh amygdalin solution in deionized water, recorded at a delay time of 6.4 µs. Labelled molecular peaks are assigned to their corresponding compounds in **Table S2**. Water peak series are partially subtracted in order to improve spectra analysis.

Spectral assignation tables

Table S1: peak assignment for LILBID cation mode spectra of amygdalin. Double colouring indicates possible contribution to the peak of two different species. Partial contribution of the pure water cluster series $[H^+(H_2O)_n]$ to certain peaks is indicated with *.

m/z	FORMULA	m/z	FORMULA
18	$[NH_4]^+$	153	$[C_5H_9O_3(H_2O)_2]^+$
29	$[CHO]^+$	157	$[C_4H_5O_2(H_2O)_4]^+$
31	$[CH_3O]^+$	159	$[C_4H_5O(H_2O)_5]^+$
36	$[NH_4(H_2O)]^+$	161	$[C_7H_7O(H_2O)_3]^+$
43	$[C_2H_3O]^+$	163*	$[GLU\text{-}OH]^+$
45	$[C_2H_5O]^+$	169	$[C_8H_7O_3(H_2O)]^+$
54	$[NH_4(H_2O)_2]^+$	170	$[MAN(H_2O)_2+H]^+$
57	$[C_3H_5O]^+/[C_2H_1O_2]^+$	171	$[C_5H_9O_3(H_2O)_3]^+$
61	$[C_2H_3O(H_2O)]^+/[C_2H_5O_2]^+$	179	$[C_7H_7O(H_2O)_4]^+$
69	$[C_4H_5O]^+$	181*	$[GLU+H]^+$
73*	$[C_3H_5O_2]^+$	187	$[C_8H_7O_3(H_2O)_2]^+$
77	$[C_6H_5]^+$	188	$[MAN(H_2O)_3+H]^+$
79	$[C_6H_7]^+$	199*	$[GLU(H_2O)+H]^+$
81	$[C_5H_5O]^+$	205	$[C_8H_7O_3(H_2O)_3]^+$
85	$[C_4H_5O_2]^+$	206	$[MAN(H_2O)_4+H]^+$
87	$[C_4H_5O(H_2O)]^+/[C_4H_7O_2]^+$	217*	$[GLU(H_2O)_2+H]^+$
91*	$[C_3H_5O_2(H_2O)]^+/[C_7H_7]^+$	223	$[C_8H_7O_3(H_2O)_4]^+$
95	$[C_6H_5(H_2O)]^+$	224	$[MAN(H_2O)_5+H]^+$
97	$[C_6H_7(H_2O)]^+/[C_5H_5O_2]^+$	235*	$[GLU(H_2O)_3+H]^+$
99	$[C_5H_7O_2]^+$	242	$[MAN(H_2O)_6+H]^+$
101	$[C_5H_9O_2]^+$	251	$[C_{12}H_{10}O_6+H]^+$
103	$[C_4H_5O_2(H_2O)]^+$	253*	$[GLU(H_2O)_4+H]^+$
105	$[C_4H_5O(H_2O)_2]^+/[C_4H_7O_2(H_2O)]^+$	271*	$[GLU(H_2O)_5+H]^+$
107	$[C_7H_7O]^+$	289*	$[GLU(H_2O)_6+H]^+$
109*	$[C_7H_7(H_2O)]^+/[GLU\text{-}(H_2O)_4+H]^+$	296	$[AMY+H\text{-}GLU\text{-}H_2O]^+$
113	$[C_6H_5(H_2O)_2]^+$	307*	$[GLU(H_2O)_7+H]^+$
115	$[C_5H_7O3]^+$	313	$[AMY+H+OH\text{-}GLU\text{-}H_2O]^+$
116	$[C_8H_6N]^+$	325*	$[2GLU\text{-}OH\text{-}H_2O]^+$
117	$[C_5H_9O_3]^+$	440	$[AMY\text{-}OH]^+$
119	$[C_5H_9O_2(H_2O)]^+$	458	$[AMY+H]^+$
121	$[C_4H_5O_2(H_2O)_2]^+$	459	[Amygdalone+H]$^+$
123	$[C_4H_5O(H_2O)_3]^+$	475	[α-Hydroxyamigdalone+H]$^+$
125	$[C_7H_7O(H_2O)]^+$	476	[Amygdalide amide+H]$^+$
127*	$[C_7H_7(H_2O)_2]^+/[GLU\text{-}(H_2O)_3+H]^+$	477	[Amygdalinic acid+H]$^+$
131	$[C_6H_5(H_2O)_3]^+$	480	[AMY+Na]$^+$
133	$[C_8H_7O_3\text{-}(H_2O)]^+$	493	[α-Hydroxyamigdalone(H_2O)+H]$^+$
134	$[MAN+H]^+$	498	[Amygdalin amide+Na]$^+$
135	$[C_5H_9O_3(H_2O)]^+$	511	[α-Hydroxyamigdalone$(H_2O)_2$+H]$^+$
139	$[C_4H_5O_2(H_2O)_3]^+$	516	[Amygdalin amide(H_2O)+Na]$^+$
141	$[C_4H_5O(H_2O)_4]^+$	534	[Amygdalin amide $(H_2O)_2$+Na]$^+$
143	$[C_7H_7O(H_2O)_2]^+$	552	[Amygdalin amide$(H_2O)_3$+Na]$^+$

**145***	$[C_7H_7(H_2O)_3]^+$/[GLU-$(H_2O)_2$+H]$^+$		**591**	[Amygdalin amide+C_8H_6N]$^+$	
**151**	$[C_8H_7O_3]^+$		**608**	[AMY+$C_8H_7O_3$]$^+$	
**152**	[MAN(H_2O)+H]$^+$				

Fragment m/z from the cation spectra are tentatively assigned to chemical formulas on **Table S1**, making use of available relevant literature.[1] Abbreviations used in **Table S1** are GLU (gentiobiose), MAN (mandelonitrile), AMY (amygdalin). Colour coding on the right-side columns indicates the major groups specified in the legend in **Figure S1**, as to better relate the contents of the table to those of **Figures S1 and S5**.

Three main groups of peaks can be set apart, occurring in the lower mass region as well as the higher mass region. Glucose and fragments arising from the two glucose rings comprise one group, together with their water clusters. Their designated colour is green. Despite presenting as a disaccharide in amygdalin, they show a similar fragmentation pattern to that of free glucose in solution [1,2].

The mandelonitrile moiety also has characteristic fragments. Mandelonitrile, its fragments and all associated water clusters, belong to the second group and are labelled with the colour orange. Finally, amygdalin and its observed derivates comprise another group, i.e. amygdalide, amygdalinic acid, amygdalone and α-hydroxyamygdalone. Together with their water and sodium clusters, they form the third group of signals and are labelled with purple and pink colours.

Table S2: peak assignment for LILBID anion mode spectra of amygdalin.

**m/z**	**FORMULA**
**431**	[Amygdalide amide-(CO_2)]$^-$
**432**	[Amygdalinic acid-(CO_2)]$^-$
**456**	[AMY-H]$^-$
**457**	[AMY]$^-$/[Amygdalone-H]$^-$
**458**	[Amygdalone]$^-$
**474**	[α-Hydroxyamigdalone]$^-$
**475**	[Amygdalide amide]$^-$
**476**	[Amygdalinic acid]$^-$
**492**	[α-Hydroxyamigdalone+(H_2O)]$^-$

Fragment m/z from the anion spectra are tentatively assigned to chemical formulas on **Table S2**. Amygdalin and amygdalone can be seen at m/z 456 and 457, respectively, as deprotonated molecular peaks. Notably, α-hydroxyamygdalone, amygdalin amide and amygdalinic acid appear at m/z 474, 475 and 476. These are not deprotonated molecular peaks, as are commonplace in anion mode LILBID spectra. Electron capture by these large molecules is more likely to play a role here, as free electrons are ubiquitous in the ion cloud, and thus these species appear as [M]$^-$. This would allow the identification of the peak at m/z 458 as [amygdalone]$^-$, and present the possibility of [AMY]$^-$ also contributing to the peak at m/z 457.

2. Amygdalone charge distribution and α carbon umpolung

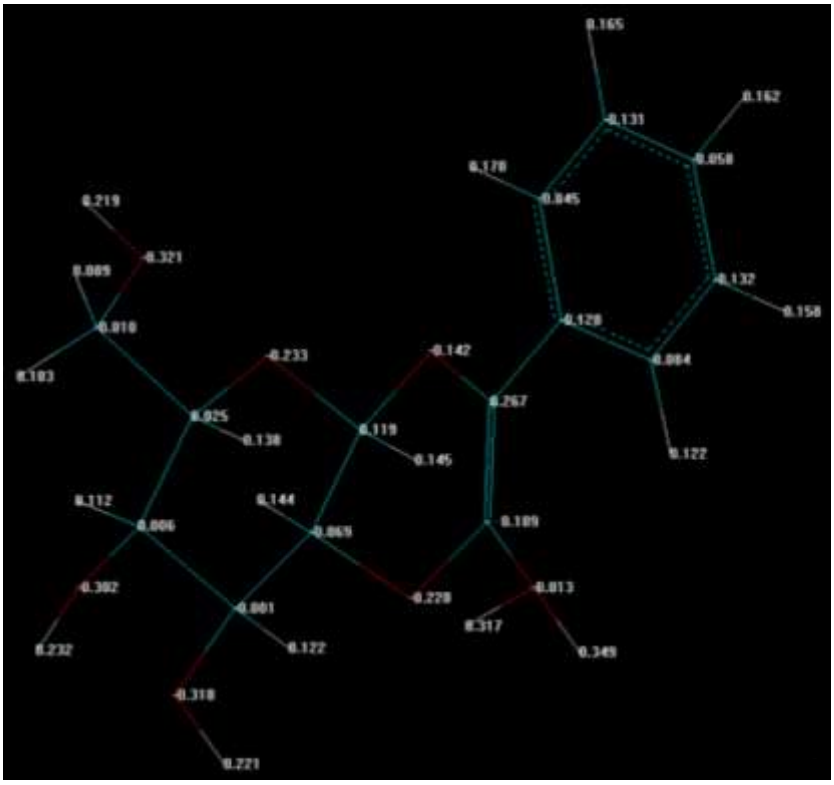

Figure S4: the structure of protonated amygdalone, computed at the semiempirical Austin Model 1 (AM1) level. This figure exhibits the stable enol form with umpolung of the α carbon, evident by the negative partial charge displayed at that position (-0.189).

3. <u>5-day old amygdalin solution cation LILBID spectra</u>

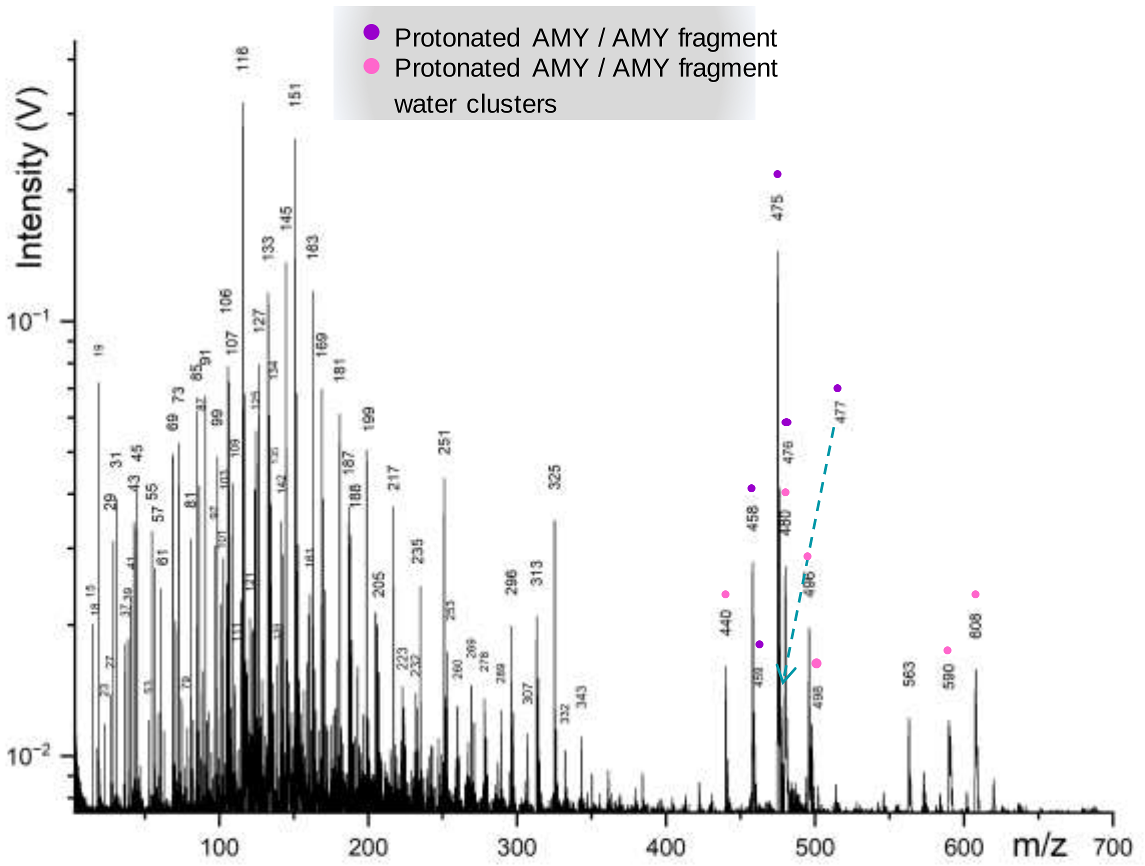

Figure S5: Baseline corrected LILBID cation mass spectra of 5 day-old-amygdalin solution in deionized water, recorded at a delay time of 6.7 µs. Peaks of amygdalin and its reaction derivates are labelled. No significant differences with cation spectra of freshly made amygdalin solution are observed.

The cation mass spectra of amygdalin recorded 5 days after solution preparation shows no significant differences in peaks to that obtained from a freshly made solution. This indicates the contents of the solution are at equilibrium. Peaks labelled are assigned to proposed molecular peaks or clusters in **Table S1**.

No anion mass spectra of the 5-day old AMY solution were recorded, as the lack of differences in the cation mass spectra of fresh and 5-day old solutions are sufficient proof of the stability of the analyte(s) in solution.

4. NMR 1D measurements

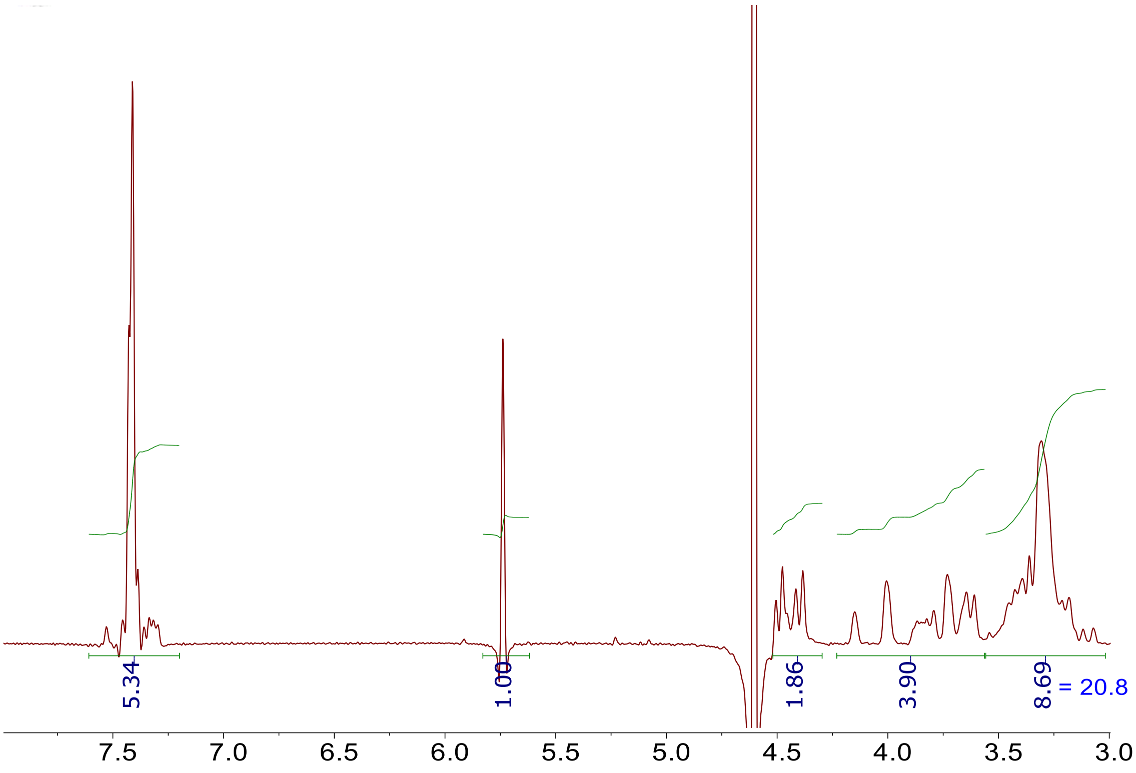

Figure S6: ^{1}H-NMR (D_2O, 80 MHz) carbon-decoupled spectrum of 4-day old amygdalin solution. The x axis represents the chemical shifts in ppm. The area under each peak is integrated and proportional to the number of nuclei (^{1}H) with the same chemical shift. Peak integrals are shown in green and integral numbers in blue. The solvent signal can be seen at 4.75 ppm, approximately.

In order to further understand the observed reactivity of amygdalin in LILBID, a second solution of amygdalin together with tosylic acid (TsOH) was measured with NMR spectrometry. TsOH is a strong organic acid, suitable for catalysing the hydrolysis of the nitrile

group in amygdalin. This particular reaction is featured as the first reaction step described for LILBID reactivity (**Figure 4**). The solution was measured immediately after addition of TsOH and 8 days later, being kept at room temperature. Both spectra, **Figure S7**, exhibit the same features as the pure amygdalin NMR spectrum, apart from the peaks corresponding to TsOH at 2.3 and 7.1 ppm: there are no appreciable significant changes in the spectral appearance of amygdalin once a strong acid is added to solution.

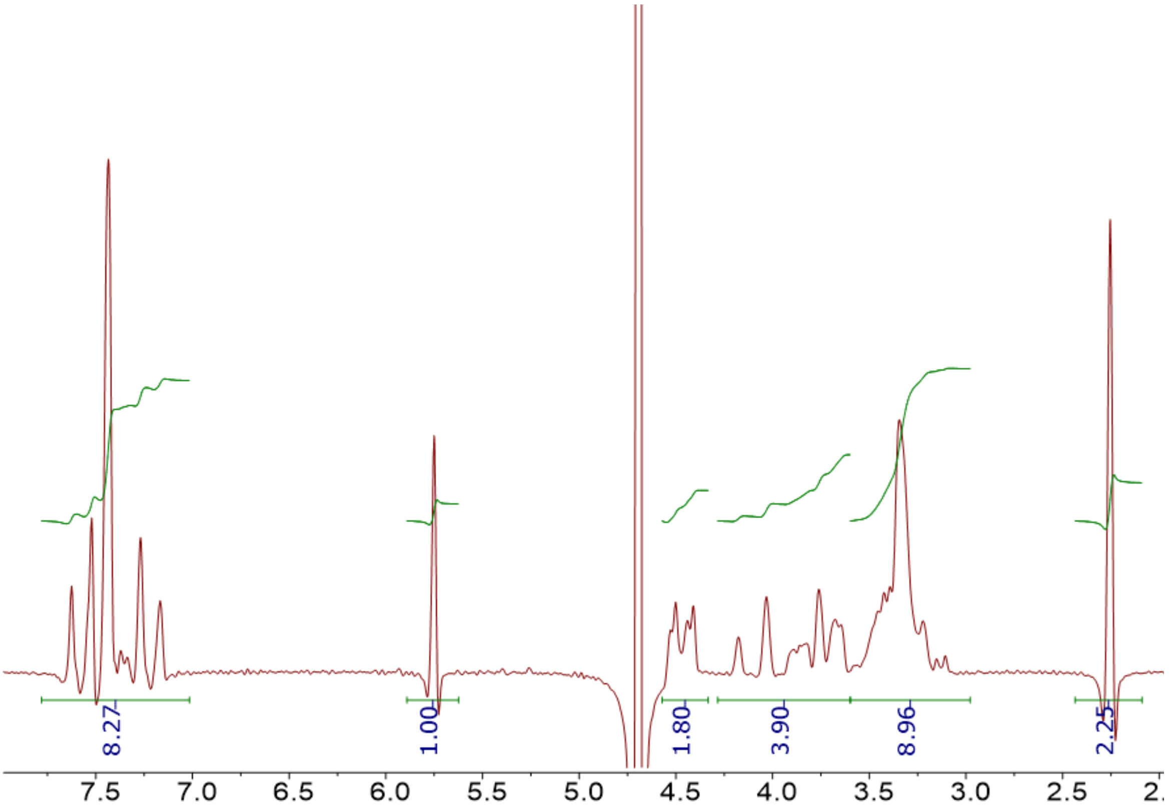

Figure S7: ^{1}H-NMR (D_2O, 80 MHz) carbon-decoupled spectrum of amygdalin/TsOH fresh solution and ca. 8 days at r.t after addition of the acid. The x axis represents the chemical shifts in ppm. The area under each peak is integrated and proportional to the number of nuclei (^{1}H) with the same chemical shift. Peak integrals are shown in green and integral numbers in blue. The solvent signal can be seen at 4.75 ppm, approximately.

The solution containing amygdalin and TsOH was then heated at 80 ºC and measured again, after 6, 72, and 144 hours, to ascertain the extent of amygdalin's stability in acidic media.

The resulting spectra show the evolution of the analytes in solution throughout the heating period, **Figures S8, S9 and S10**. The spectra in **Figure S11** conveys the clear differences when comparing ^{1}H-NMR spectra of amygdalin recorded prior to heating and after heating.

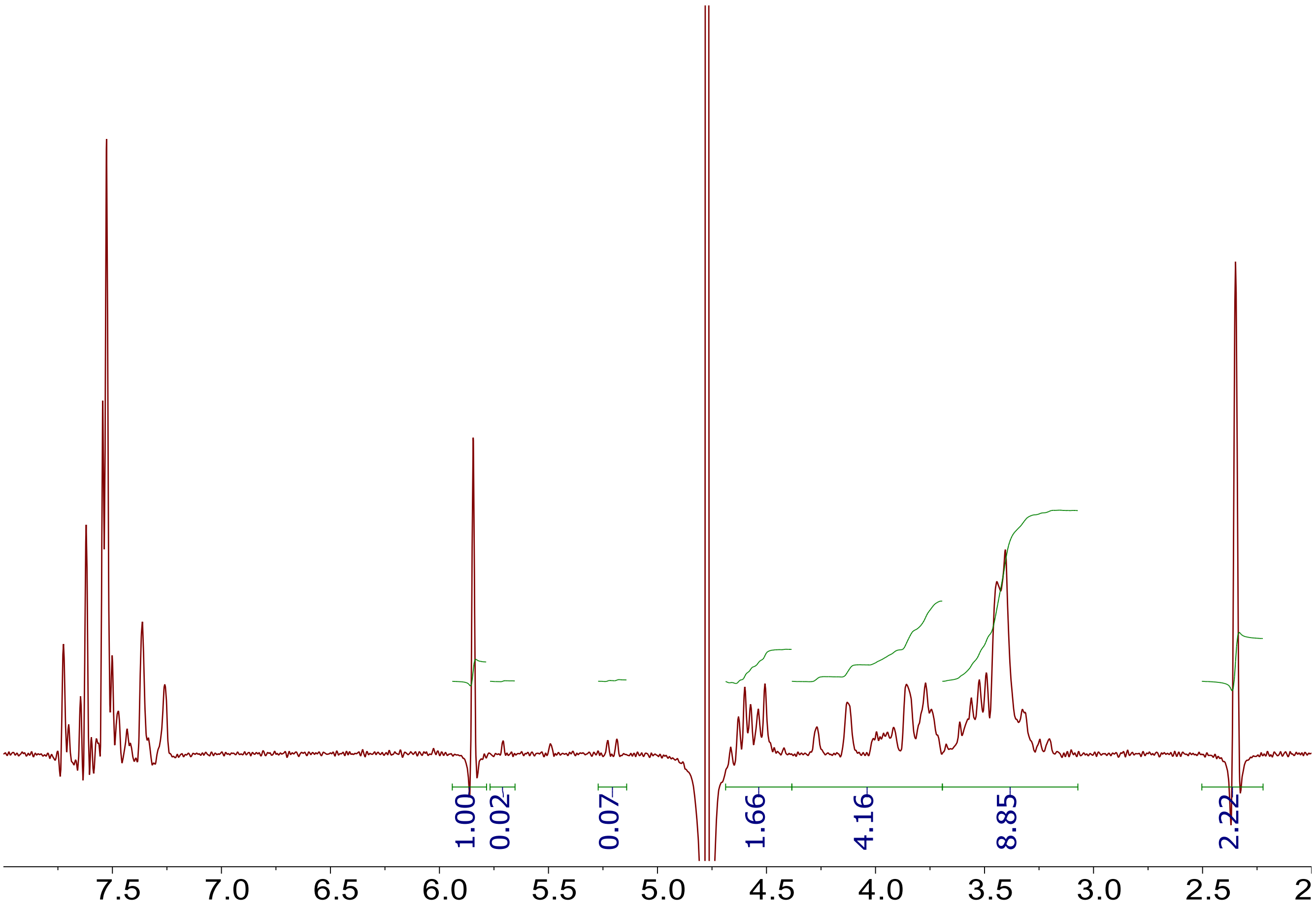

Figure S8: ^{1}H-NMR (D_2O, 80 MHz) carbon-decoupled spectrum of amygdalin/TsOH fresh solution and ca. 8 days at r.t and 6 h at 80 ºC. The x axis represents the chemical shifts in ppm. The area under each peak is integrated and proportional to the number of nuclei (^{1}H) with the same chemical shift. Peak integrals are shown in green and integral numbers in blue. The solvent signal can be seen at 4.75 ppm, approximately.

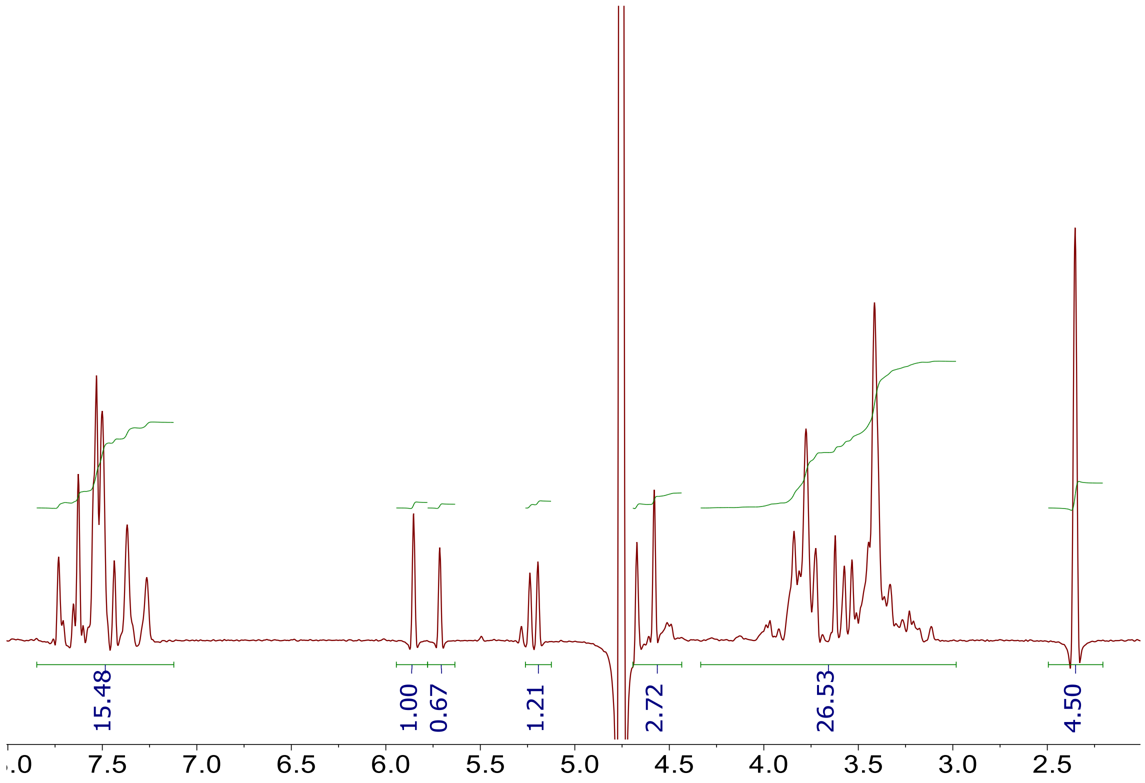

Figure S9: ^{1}H-NMR (D_2O, 80 MHz) carbon-decoupled spectrum of amygdalin/TsOH fresh solution and ca. 8 days at r.t and 72 h at 80 °C. The x axis represents the chemical shifts in ppm. The area under each peak is integrated and proportional to the number of nuclei (^{1}H) with the same chemical shift. Peak integrals are shown in green and integral numbers in blue. The solvent signal can be seen at 4.75 ppm, approximately.

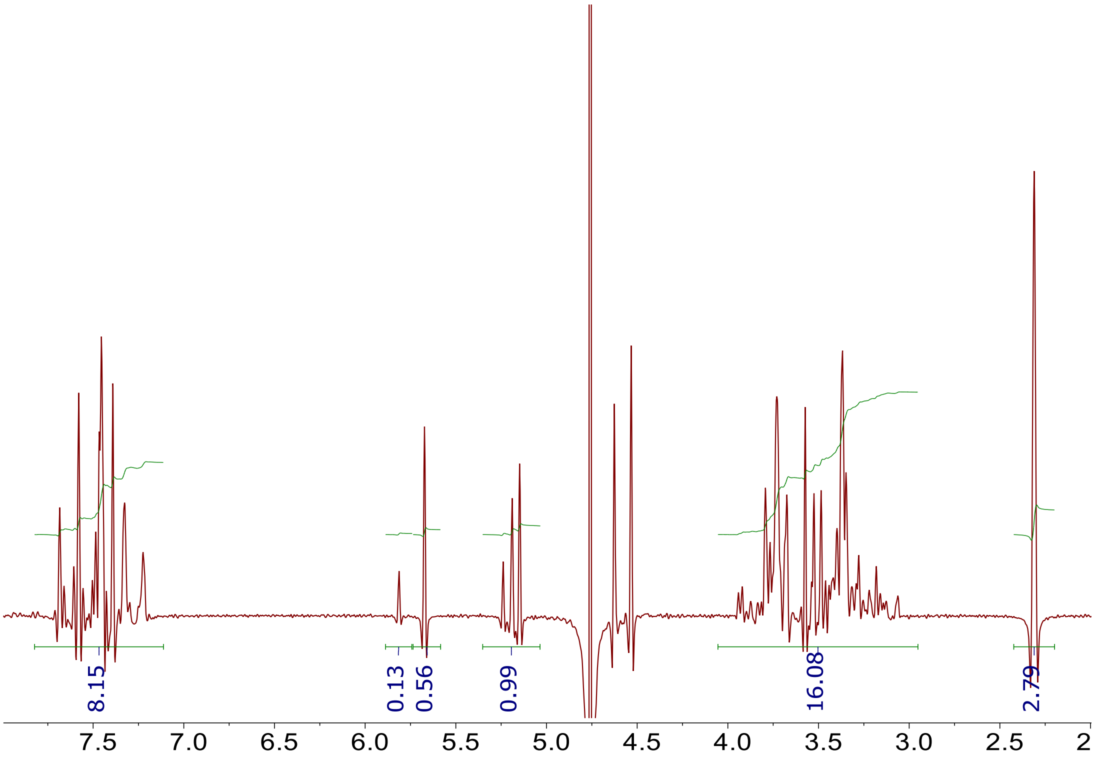

Figure S10: ^{1}H-NMR (D_2O, 80 MHz) carbon-decoupled spectrum of amygdalin and TsOH in solution after 144 h at 80 ºC. The x axis represents the chemical shifts in. The area under each peak is integrated and proportional to the number of nuclei (^{1}H) with the same chemical shift. Peak integrals are shown in green and integral numbers in blue. The solvent signal can be seen at 4.75 ppm, approximately.

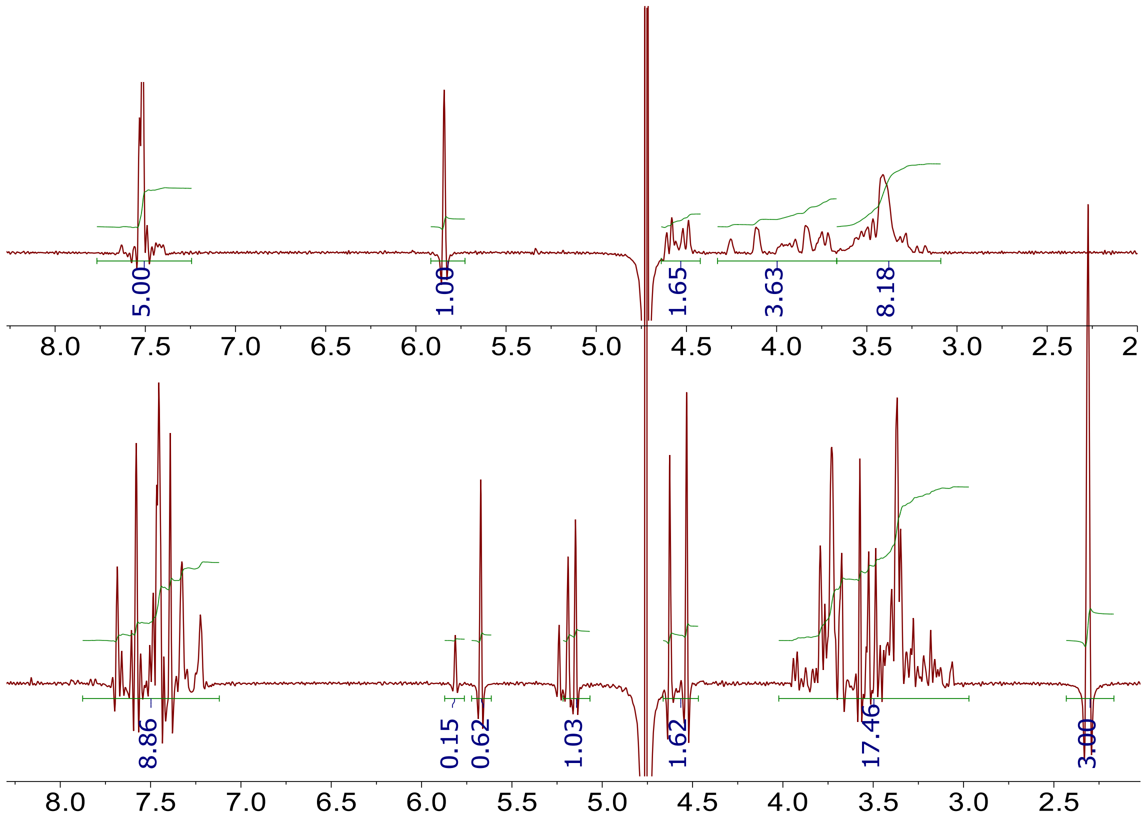

Figure S11: comparison of ^{1}H-NMR (D_2O, 80 MHz) carbon-decoupled spectra in **Supporting Information S6** (above) and **Figure S10** (below). The x axis represents the chemical shifts in ppm. The area under each peak is integrated and proportional to the number of nuclei (^{1}H) with the same chemical shift. Peak integrals are shown in green and integral numbers in blue. The solvent signal can be seen at 4.75 ppm, approximately.

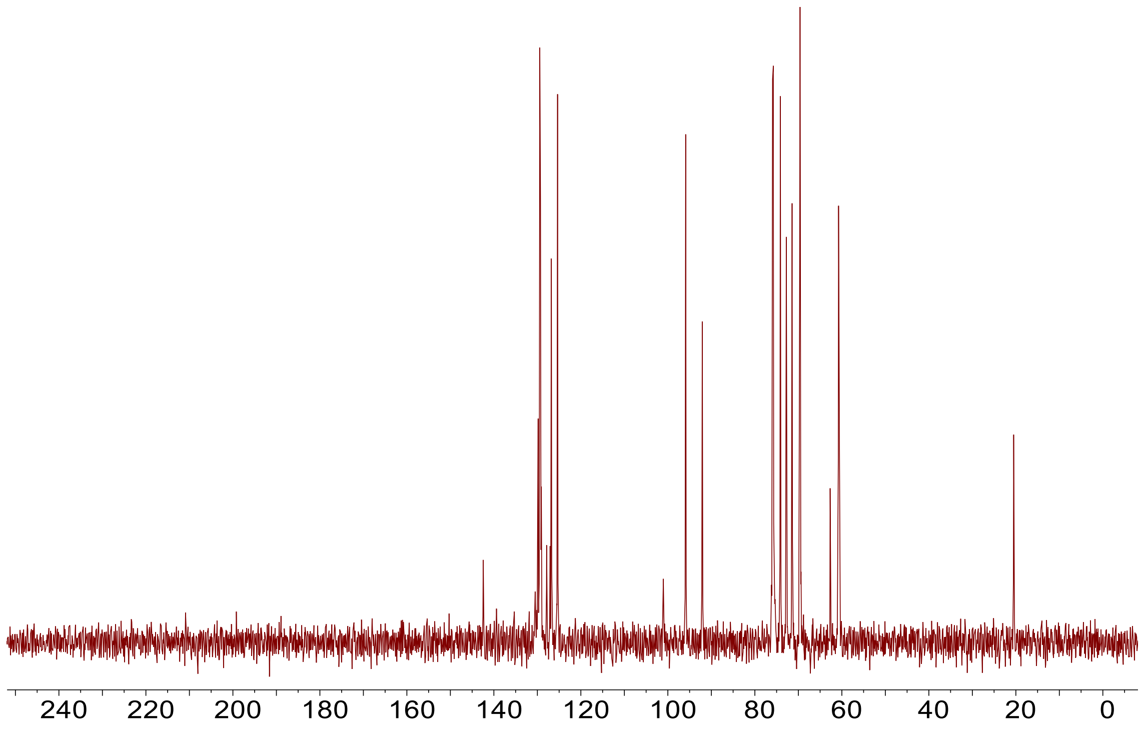

Figure S12: ^{13}C-NMR (D2O, 20 MHz) proton-decoupled spectrum of amygdalin/TsOH solution after 144 h at 80 ºC. The x axis represents the chemical shifts in ppm.

Notably, no peaks in the 170-190 ppm region appear, indicating the absence of carboxylic derivatives in solution. Further work was carried out in order to identify other disparities between reactivity in the gas phase and in the liquid phase, as degradation products of amygdalin in acidic media are not the same as those identified in the main body of this work.

^{1}H-NMR and ^{13}C-NMR spectra of glucose were obtained in similar measuring conditions (**Figures S13 and S14**), alongside a ME-HSQC $^{1}H/^{13}C$ spectrum of the decomposition product of amygdalin in solution (**Figure S15**), and glucose in solution (**Figure S16**). These spectra provide valuable insight as to the fate of amygdalin in solution with TsOH when heat is applied, as they hint at the fractionation of amygdalin into its constituent moieties: glucose and mandelonitrile.

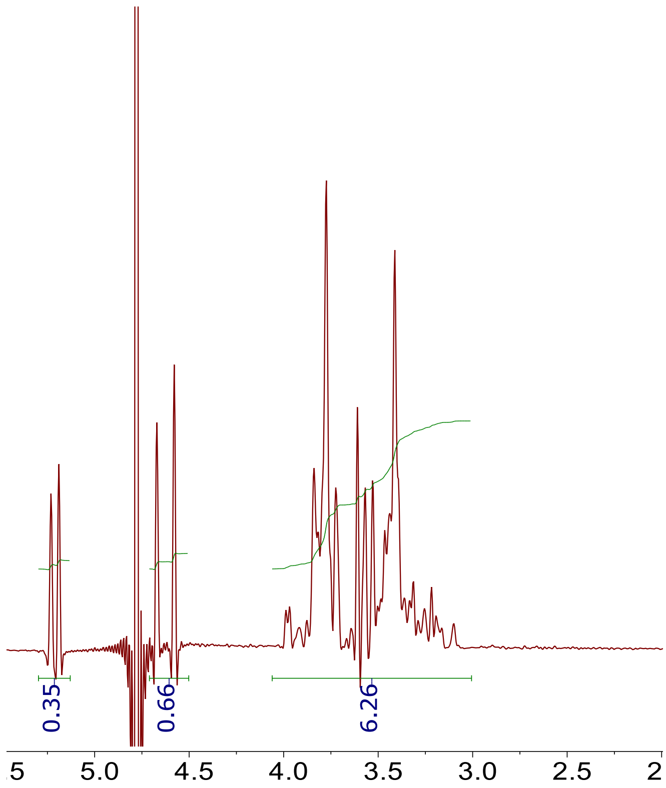

Figure S13: ^{1}H-NMR (D_2O, 80 MHz) carbon-decoupled spectrum of glucose. The x axis represents the chemical shifts in ppm. The area under each peak is integrated and proportional to the number of nuclei (^{1}H) with the same chemical shift. Peak integrals are shown in green and integral numbers in blue. The solvent signal can be seen at 4.75 ppm, approximately.

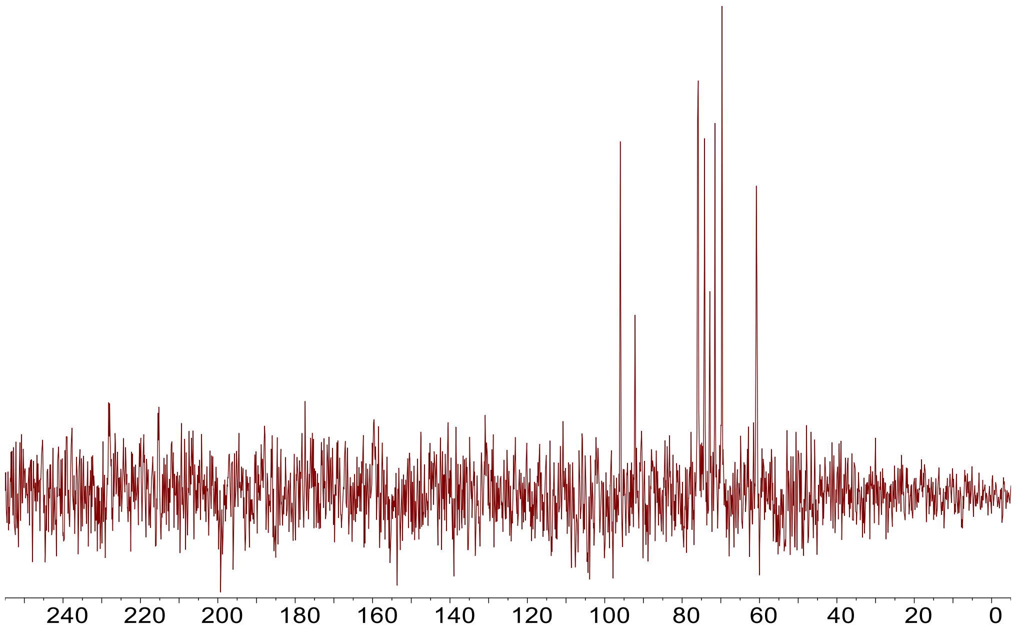

Figure S14: ^{13}C-NMR (D_2O, 80 MHz) proton-decoupled spectrum of glucose. The x axis represents the chemical shifts in ppm.

5. <u>NMR 2D measurements</u>

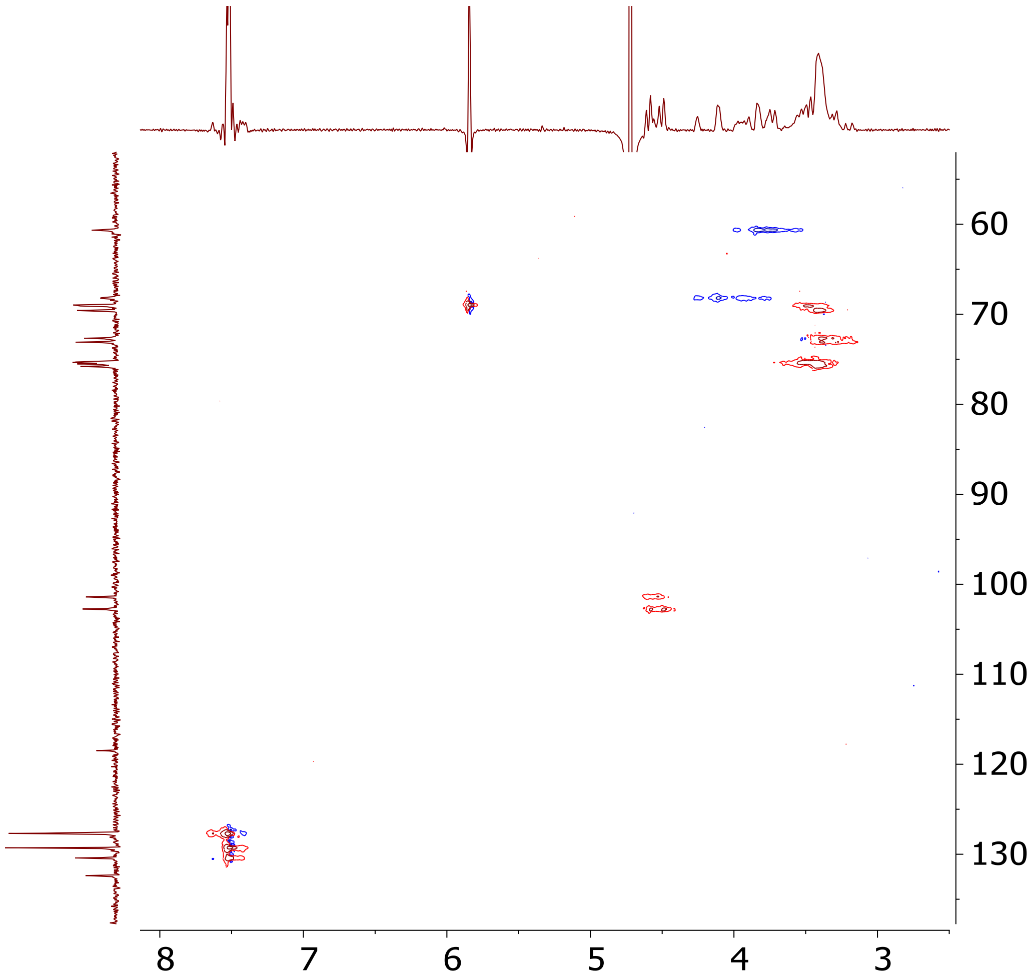

Figure S15: ME-HSQC $^1H/^{13}C$ (D_2O, 80 MHz) proton-detected 2D correlation spectrum of amygdalin in solution. The axes down and right represent the chemical shift with units of ppm. The intensity of shade at a given coordinate indicates peak correlation.

Figure S15 shows the ME-HSQC 1H/13C spectrum for amygdalin, correlating proton and carbon atoms directly bonded. It confirms the assignment done in section 4 of the Supporting Information, relating the signal at 6 ppm in the proton spectra with a carbon signal reflecting both the electron donating effect of the adjacent oxygen as well as the electron withdrawing effect of the nitrile group, appearing in the spectrum's mid-range. It also relates the acetal

protons to their carbons around 100 ppm as well as the aromatic protons to their carbons around 130 ppm.

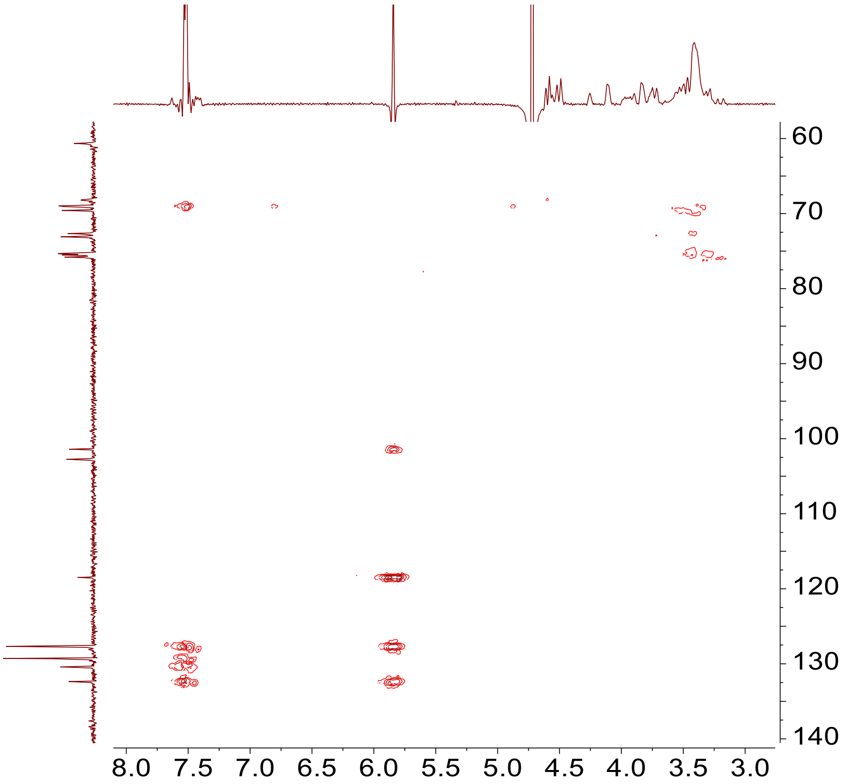

Figure S16: HMBC ^{1}H /^{13}C (D_2O, 80 MHz) proton-detected 2D correlation spectrum of amygdalin in solution. The axes down and right represent the chemical shift with units of ppm. The intensity of shade at a given coordinate indicates peak correlation.

Figure S16 shows the HMBC ^{1}H /^{13}C spectrum for amygdalin and gives correlations between carbons and protons that are separated by two or three bonds. Thus, the proton in the benzylic position, at 6 ppm, "sees" the carbons of the adjacent acetal group as well as the phenylic protons and the nitrile's carbon (120 ppm).

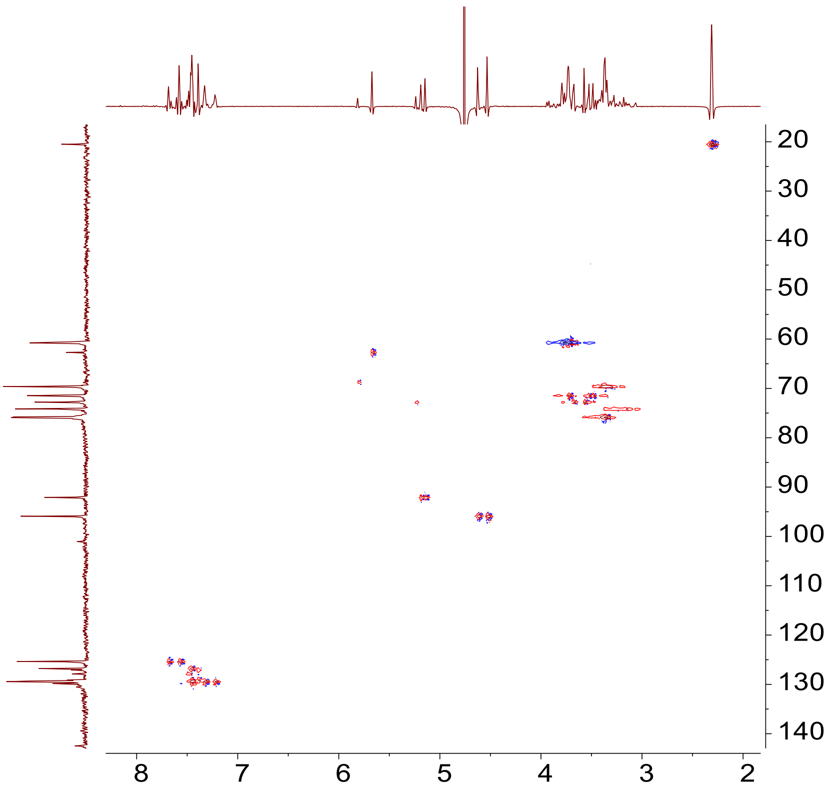

Figure S17: ME-HSQC $^{1}H/^{13}C$ (D_2O, 80 MHz) proton-detected 2D correlation spectrum of the decomposition product of amygdalin in solution treated with TsOH and heat (144 h). The axes down and right represent the chemical shift with units of ppm. The intensity of shade at a given coordinate indicates peak correlation.

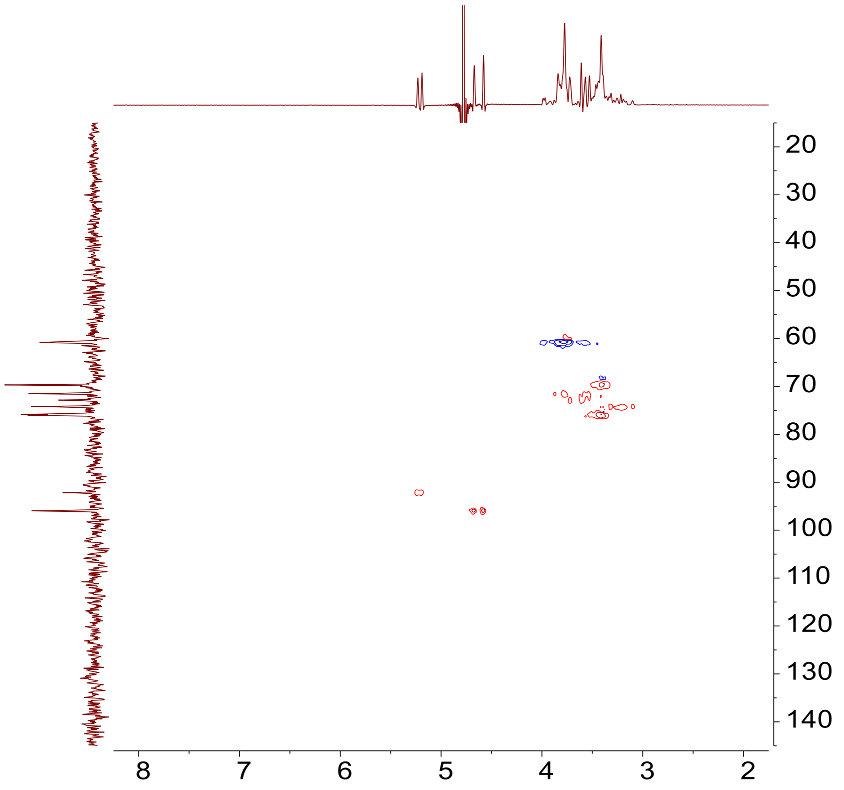

Figure S18: ME-HSQC $^{1}H/^{13}C$ (D_2O, 80 MHz) proton-detected 2D correlation spectrum of glucose. The axes down and right represent the chemical shift with units of ppm. The intensity of shade at a given coordinate indicates peak correlation.

Comparison of the spectra in **Figure S17** to that of pure glucose (**Figure S18**) indicates amygdalin is broken up into its constituent: glucose and mandelonitrile, when heated in acid media. Other studies of amygdalin hydrolysis in acid media also describe this reaction pathway.[3]

6. NMR amygdalin chemical shift assignation

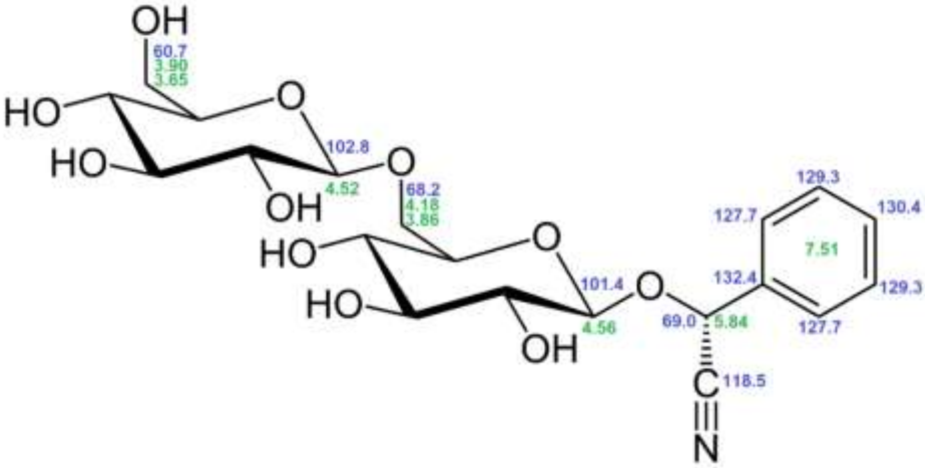

Figure S19: structure of amygdalin with partially assigned chemical shifts for carbon (blue) and proton (green) positions, from NMR ^{1}H-NMR and ^{13}C-NMR spectra (**Figures 6 and 7**), relevant for structural elucidation.

Bibliography:

1. Napoleoni, M.; Klenner, F.; Khawaja, N.; Hillier, J. K.; Postberg, F. “Mass Spectrometric Fingerprints of Organic Compounds in NaCl-Rich Ice Grains from Europa and Enceladus.” *ACS Earth Space Chem*. 2023, 7 (4), 735–752. https://doi.org/10.1021/acsearthspacechem.2c00342.
2. Napoleoni, M.; Klenner, F.; Hortal Sánchez, L.; Khawaja, N.; Hillier, J. K.; Gudipati, M. S.; Hand, K. P.; Kempf, S.; Postberg, F. “Mass Spectrometric Fingerprints of Organic Compounds in Sulfate-Rich Ice Grains: Implications for Europa Clipper.” *ACS Earth Space Chem. 2023*, 7 (9), 1675–1693. https://doi.org/10.1021/acsearthspacechem.3c00098.

3. Walker, J. W. & Krieble, V. K. "The hydrolysis of amygdalin by acids. Part I" *J. Chem. Soc., Trans.* 1909, 95, 1369-1377.
